# Piperazine-grafted magnetic graphene oxide as a sustainable heterogeneous catalyst for solvent-free Biginelli reaction

**DOI:** 10.1039/d5ra05063d

**Published:** 2026-02-24

**Authors:** Esmail Rezaei-Seresht, Faezeh Jalambadani, Samira Cheshak, Behnam Mahdavi, Fatemeh Tafazzoli Gazkoh

**Affiliations:** a Department of Chemistry, School of Sciences, Hakim Sabzevari University Sabzevar Iran e.rezaei@hsu.ac.ir +98 5144013516

## Abstract

Multi-component reactions (MCRs) are powerful tools for the one-pot synthesis of complex organic compounds, with the Biginelli reaction being one of the most important due to its versatility in synthesizing a wide range of dihydropyrimidine-2-ones (DHPMs) with diverse biological activities. However, traditional MCR catalysts are often homogeneous and challenging to recycle, leading to environmental concerns and high costs. In this study, we developed a novel heterogeneous catalyst for the Biginelli reaction by functionalizing graphene oxide (GO) with piperazine (NH) to form a Fe_3_O_4_@GO-NH nanocomposite. The catalyst was characterized using FT-IR, TGA, and SEM/EDX and showed high catalytic activity for the Biginelli reaction under solvent-free conditions. The optimal reaction conditions were determined by adjusting various parameters such as temperature, stability and reproducibility, time, and the molar percentage of Fe_3_O_4_@GO-NH. As a result of this eco-friendly approach, 32 Biginelli products were successfully obtained with high yields. The catalyst was also reusable, making it a more environmentally friendly and cost-effective alternative to conventional homogeneous catalysts.

## Abstract 2

Biginelli reaction is a powerful tool for the one-pot synthesis of complex organic compounds, due to its versatility in synthesizing a wide range of dihydropyrimidine-2-ones (DHPMs) with diverse biological activities.

## Graphical abstract

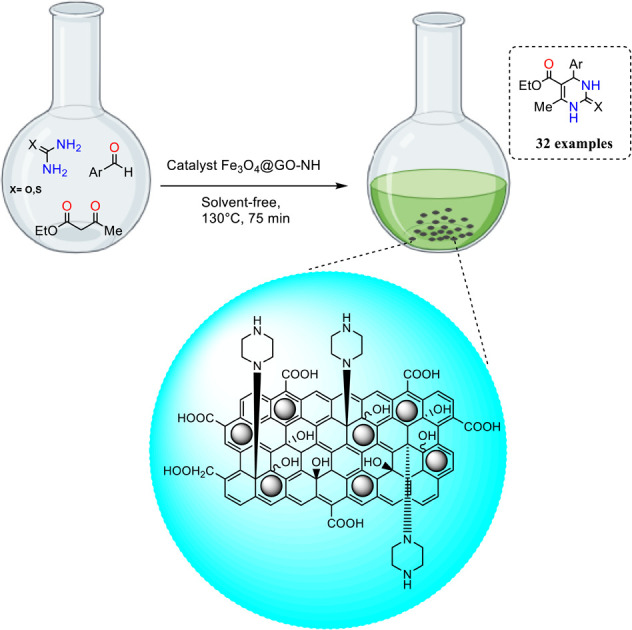

## 1. Introduction

Multi-component reactions (MCRs) are a class of synthetic methods in which several raw materials are mixed to form a one-pot product.^1^ In these reactions, according to the conditions and the nature of the chemicals involved in the reaction, all the components in MCRs may react linearly (such as the Mannich reaction) or cyclize to form more complex molecules (such as the Hantzsch dihydropyridine synthesis).^2^ MCRs, characterized by their unique one-step nature, offer economic advantages by eliminating intermediate product separation and purification while achieving higher yields than two- or multi-step reactions.^3,4^ A prime example of a versatile MCR is the Biginelli reaction, discovered in 1893.^3,4^ This three-component condensation reaction efficiently produces dihydropyrimidinones (DHPMs), a class of molecules exhibiting diverse biological activities such as antiviral, anti-tumor, antibacterial, and anti-inflammatory properties.^5–10^ DHPMs are being progressively utilized to create materials like polymers, adhesives, fabric dyes, pharmaceutical compounds, and more.^5^ The Biginelli reaction typically involves a 1,3-dicarbonyl compound, an aromatic aldehyde, and urea/thiourea under acidic conditions.^11,12^ The DHPMs derived from the Biginelli reaction have attracted significant interest in drug discovery due to their potential therapeutic applications. Current research focuses on developing DHPM-based drugs for various diseases, including neurodegenerative, cardiovascular, and metabolic disorders.^13–16^

While the Biginelli reaction is well-established, ongoing efforts seek to optimize its efficiency and sustainability. Traditional methods often involve harsh acidic conditions, long reaction times, and toxic solvents. In addition, most reported catalysts for the Biginelli reaction are homogeneous acids or metal salts, suffering from drawbacks such as difficulty in separation and recycling, catalyst leaching, and generation of toxic waste. In some cases, the reported heterogeneous catalysts showed limited activity, required high temperatures, or produced moderate yields due to poor active site accessibility or low surface area.^17–20^ Researchers are actively developing new catalysts and reaction conditions to address these challenges, aiming for greener and more efficient syntheses of DHPMs.^21–25^

Graphene oxide (GO), a single layer of sp^2^ hybridized carbon atoms arranged in a honeycomb lattice structure, possesses oxygen-containing functional groups such as hydroxyl, epoxide, and carboxyl groups.^6^ These functional groups endow GO with unique properties that make it an ideal candidate for catalytic applications in organic synthesis. Notably, GO boasts a high surface area, facilitating the adsorption of various reaction components.^7^ Additionally, the oxygen functionalities on GO impart Brønsted acidity, enabling it to act as a proton donor and participate in reaction mechanisms.^8^ These features collectively position GO as a versatile platform for developing efficient and sustainable catalysts for MCRs. GO has emerged as a versatile catalyst for various MCRs. Its unique properties, including Brønsted acidity, bio-compatibility, high surface area, inertness, and outstanding electronic, optical, thermal and mechanical properties, improve reaction efficiency across diverse MCR types.^9–11^ Moreover, it is derived from affordable and readily available starting materials. One notable derivative of GO is GO-NH, which is obtained through the functionalization of GO with piperazine molecules^12^ This functionalization process involves the nucleophilic attack of piperazine on the epoxide carbons present on the GO sheet, leading to the opening of the epoxide rings and the attachment of piperazine molecules onto the GO surface. In the Biginelli reaction, for example, GO's unique combination of acidity and adsorption properties is believed to significantly improve yields and reaction times for synthesizing biologically active DHPMs compared to traditional methods.^5^ This trend continues with the Knoevenagel condensation, a versatile MCR for creating valuable α,β-unsaturated carbonyl compounds. Here, π–π stacking interactions between GO and aromatic aldehydes are thought to be crucial in boosting reaction efficiency.^6^ GO's effectiveness further benefits the Mannich reaction, a three-component condensation for β-amino carbonyl compounds.^7^

To address the mentioned catalyst drawbacks in the Biginelli reaction, such as limited activity, difficulty in separation and recycling, low surface area, the generation of toxic waste, and the use of toxic solvents in the reaction. We envisaged that composite Fe_3_O_4_@GO-NH has several features that make it a promising catalyst for reactions involving multiple proton-transfer (protonation and deprotonation) steps, such as the Biginelli reaction. The Fe_3_O_4_ particles provide Lewis acid sites to activate the aldehyde. At the same time, the piperazine functionalization introduces basic sites that enhance the nucleophilicity of urea (or thiourea) and facilitate enolate formation from the β-keto ester component. Traditional catalysts often fail to manage these dual roles efficiently, leading to lower yields. Additionally, the GO component offers a high surface area, improving catalytic activity. Finally, the composite's magnetic properties enable easy separation and recycling, making it an efficient and reusable catalyst.

Therefore, this study focuses on preparing and characterizing Fe_3_O_4_@GO-NH and its application as a heterogeneous, magnetically-separable catalyst in the solvent-free Biginelli reaction for synthesizing dihydropyrimidine-2-ones.

## 2. Experimental

### 2.1. Materials and reagents

#### 2.1.1. Reagents

All chemicals were purchased from Merck and used without further purification. Precoated silica gel 60 F254 plates (Merck) were used for TLC detection.

#### 2.1.2. Catalyst preparation (Fe_3_O_4_/GO-NH)

As described previously, catalyst Fe_3_O_4_@GO-NH was synthesized using a method based on the procedure outlined by Cid *et al.*^8^ Initially, GO-NH (0.30 g) was suspended in distilled water (200 mL). Then, FeCl_2_·4H_2_O (2.0 g, 10 mmol) and FeCl_3_·6H_2_O (4.0 g, 14.8 mmol) were added to this suspension. The mixture was stirred at 50 °C for 3 hours. Following this, ammonia (10 mL) was added dropwise to achieve a pH of 11, and stirring continued for an additional hour at the same temperature. Finally, the synthesized Fe_3_O_4_@GO-NH was magnetically separated, washed several times with distilled water and ethanol, and dried in a vacuum at 80 °C.^9^ The successful attachment of piperazine was confirmed by elemental analysis, which revealed a nitrogen content of 6.46 wt% for GO-NH.

### 2.2. Instrumental

^1^H NMR spectra were taken by Bruker Advance-300 MHz in DMSO-*d*_6_ solvent. The FT-IR 8400s device made by SHIMADZU Company was used to prepare the IR spectrum of synthetic products by KBr tablets in the 400–4000 cm^−1^ frequency range. An Electrothermal-9200 device was used to determine the melting point of the products. In order to check the progress of the reaction, the purity and identification of the compounds were performed by TLC chromatography using a UV lamp with a wavelength of 256–365 made by Merck. Also, the Fe_3_O_4_@GO-NH catalyst was identified by the EDX device made by BuAli Institute with an accelerating voltage of 150 kV, a beam current of 4 700 000 000 nA and a magnification of 100 000. The XRD pattern was obtained with Cu Kα radiation at 45 kV and 40 mA and at a rate of 2° min^−1^, using the STOE PW2773.00 device. Elemental analysis was performed on a TRUSPEC CHNS analyzer.

### 2.3. Catalytic experiments

#### 2.3.1. Experimental method for Biginelli reaction (synthesis of compounds 1–32)

A mixture of aromatic aldehyde (1 mmol), ethyl acetoacetate (1 mmol), urea and thiourea (1.3 mmol) and catalyst Fe_3_O_4_@GO-NH (0.015 g, 11.5 wt% loading) was heated under solvent-free conditions and at a temperature of 130 °C for 75 min. The progress of the reaction was monitored by TLC (ethyl acetate : *n*-hexane 1 : 1). Finally, the reaction mixture was cooled at room temperature and then dissolved in 5 mL of ethanol, and after separating the catalyst by an external magnet, the remaining liquid was poured into ice-water (50 mL), and the precipitate obtained was washed with water and dried.

#### 2.3.2. Representative spectral data for the Biginelli products

##### 2.3.2.1 Ethyl 4-(4-bromophenyl)-6-methyl-2-oxo-1,2,3,4-tetrahydropyrimidine-5-carboxylate (2b)

Light yellow powder (0.33 g, 98%); mp 215 °C (from ethanol) (lit., 15 215 °C); IR (KBr) *v*_max_/cm^−1^ 3340 and 3120 (N–H), 1723 (C=O), 1550 (C=C), 1468 (CH_2_), 1383 (CH_3_), 1240 (C–O); ^1^H NMR *δ*_H_ (300 MHz; DMSO-*d*_6_; Me_4_Si) 1.11 (3H, t, *J* = 6.3 Hz, OCH_2_*CH*_*3*_). 2.26 (3H, s, CH_3_), 4.01 (2H, q, *J* = 6.3 Hz, O*CH*_*2*_CH_3_), 5.14 (1H, s, CH), 7.21 (2H, d, *J* = 6.9 Hz, H-aromatic), 7.47 (2H, d, *J* = 6.9 Hz, H-aromatic), 7.79 (1H, s, NH), 9.26 (1H, s, NH).

##### 2.3.2.2 Ethyl 4-(4-chlorophenyl)-6-methyl-2-oxo-1,2,3,4-tetrahydropyrimidine-5-carboxylate (2c)

White powder (0.29 g, 97%); mp 210 °C (from ethanol) (lit., 16 212 °C); IR (KBr) *v*_max_/cm^−1^ 3318 and 3133 (N–H), 1722 (C=O), 1575 (C=C), 1443 (CH_2_), 1314 (CH_3_), 1174 (C–O), 710 (C–Cl); ^1^H NMR *δ*_H_ (300 MHz; DMSO-*d*_6_; Me_4_Si) 1.11 (3H, t, *J* = 6.9 Hz, OCH_2_*CH*_*3*_). 2.27 (3H, s, CH_3_), 4.01 (2H, q, *J* = 6.9 Hz, O*CH*_*2*_CH_3_), 5.15 (1H, d, *J* = 3.0 Hz CH), 7.27 (2H, d, *J* = 7.8 Hz, H-aromatic), 7.41 (2H, d, *J* = 7.8 Hz, H-aromatic), 7.79 (1H, s, NH), 9.27 (1H, s, NH).

#### 2.3.3. Recovery of Fe_3_O_4_@GO-NH

Catalyst Fe_3_O_4_@GO-NH was isolated from the reaction mixture using an external magnet. Multiple washes were performed with ethanol and acetone to purify and decontaminate the catalyst. Subsequently, the sample was dried in a vacuum oven at 100 °C for 1 hour.

#### 2.3.4. PZC determination of Fe_3_O_4_@GO-NH and Fe_3_O_4_@rGO-NH

Equilibrium experiments were carried out at room temperature (25 ± 2 °C) using 0.01 M KCl as the background electrolyte to maintain constant ionic strength. Increasing masses of the colloidal material (Fe_3_O_4_@GO-NH) were added to a fixed volume (50 mL) of the electrolyte solution, and the suspensions were allowed to equilibrate under stirring for 24 h to ensure complete mixing and ion exchange. The equilibrium pH of each suspension was then measured, and the PZC was identified as the corresponding pH to the plateau region in the curve of the pH *vs.* mass-to-volume ratio (Fig. S8 and S9 in SI).

## 3. Results and discussion

### 3.1. Synthesis and characterization of composite Fe_3_O_4_@GO-NH

**Scheme 1:**
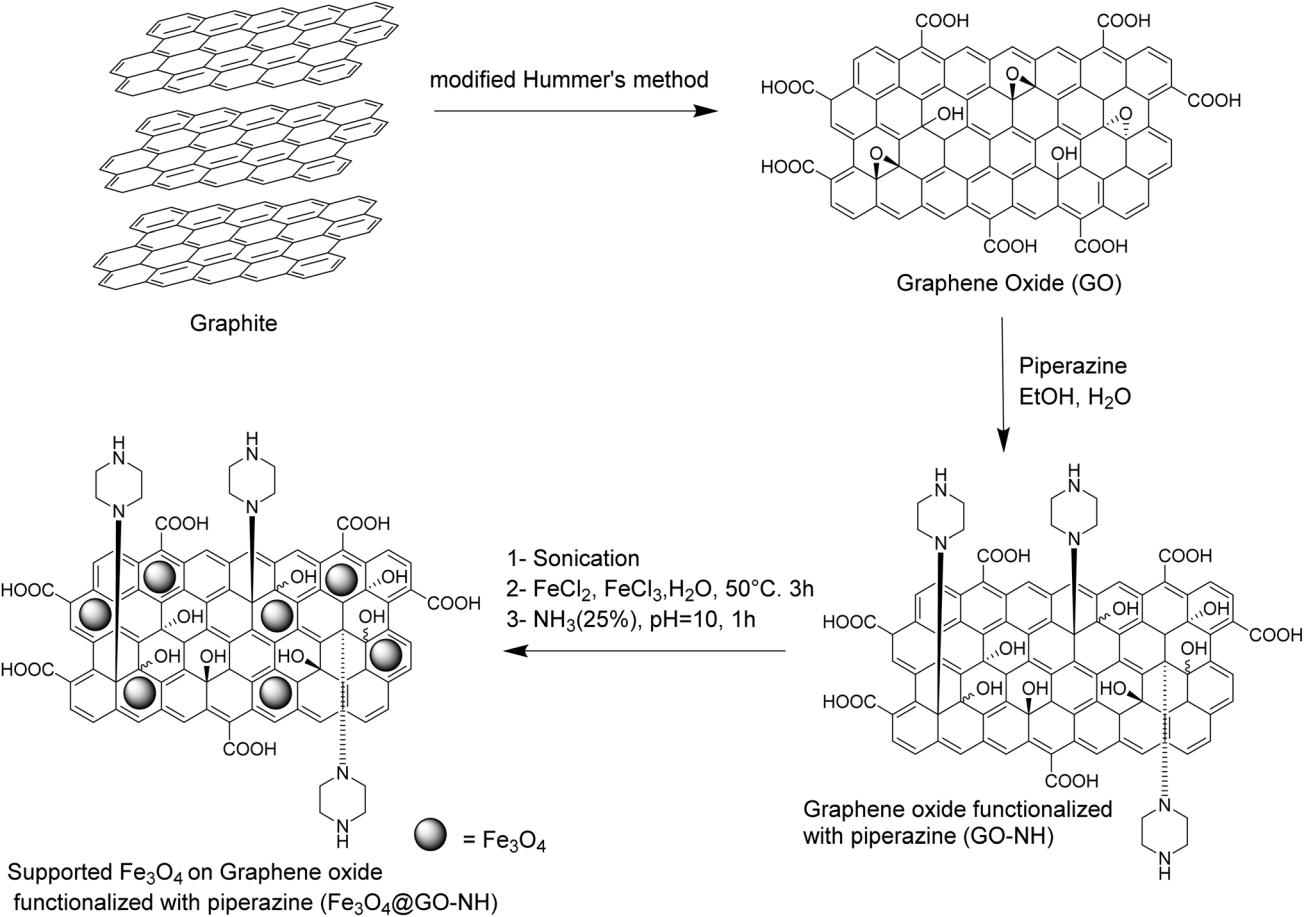
Synthesis of catalyst Fe_3_O_4_@GO-NH.

Initially, GO was synthesized from graphite using a modified version of Hammer's method.^10^ Subsequently, GO was functionalized with piperazine molecules (GO-NH) through a nucleophilic attack on the epoxide carbons present on the GO sheet.^11^ Elemental analysis of GO-NH revealed a nitrogen content of 6.46 wt%, indicating the presence of piperazine molecules. Finally, the magnetic catalyst Fe_3_O_4_@GO-NH was synthesized *via* the co-precipitation method, as outlined in Scheme 1. The characterization of the catalyst was confirmed using various techniques, including TGA and EDX.

The thermogravimetric analysis (TGA) of the catalyst (SI) shows two main steps of weight loss, which are important for assessing its stability at the reaction temperature of 130 °C. The first weight loss (∼32%) occurs in the range of approximately 28–92 °C and is attributed to the removal of moisture and volatile surface species. The weight change between 92 °C and 130 °C is minimal (a minor weight loss of ∼2% between 91 and 130 °C). This slight loss is attributed to the evaporation of surface-adsorbed water, which is consistent with the hydrophilic nature of the GO sheets. Therefore, the catalyst retains its structural integrity and chemical stability under the reaction conditions (130 °C). The elemental composition of catalyst Fe_3_O_4_@GO-NH were determined by EDX (SI). Peak N Kα belongs to nitrogen, suggesting a notable amount of nitrogen content in the catalyst. Also, several peaks associated with iron (Fe Lα, Fe Kα, and Fe Kβ) are visible, indicating the presence of iron in the catalyst. Therefore, the EDX spectrum suggests that the catalyst primarily contains elements iron, oxygen, nitrogen and carbon in its composition.

To get an insight on the nature of acid–base sites of Fe_3_O_4_@GO-NH that are essential in catalysis of the Biginelli reaction, the point of zero charge (PZC) for catalyst Fe_3_O_4_@GO-NH was determined by mass titration method described by Noh and Schwarz.^12^ A PZC value of 4.70 (SI) suggests that the Fe_3_O_4_@GO-NH surface is net positively charged at pH < 4.7 and negatively charged above this value. This behavior reflects the coexistence of acidic sites derived from –COOH groups on the GO surface and basic sites from piperazine grafting.

### 3.2. Catalytic properties of Fe_3_O_4_@GO-NH in model reaction

**Scheme 2:**
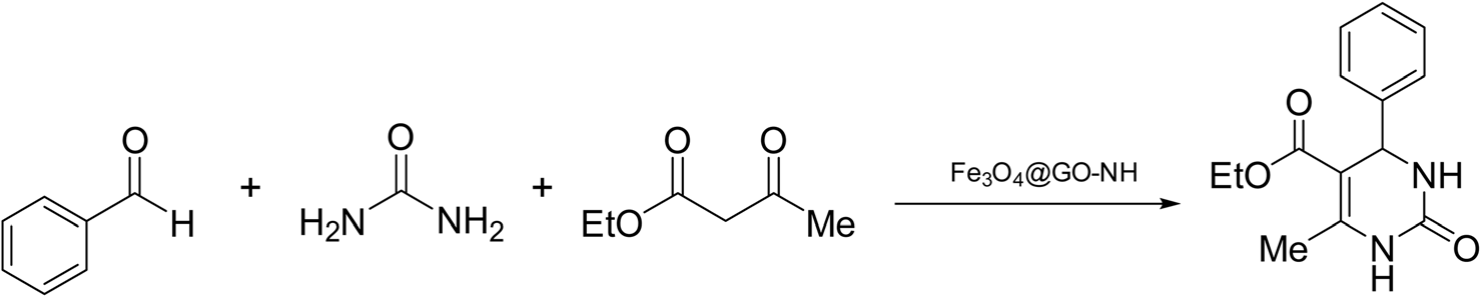
Schematic representation of the model reaction.

We chose the reaction of benzaldehyde (1 mmol), urea (1.3 mmol) and ethyl acetoacetate (1,3-dicarbonyl) (1 mmol) as the model reaction to evaluate the catalytic efficiency of Fe_3_O_4_@GO-NH in the Biginelli reaction, as well as to achieve optimal reaction parameters, including temperature, time, amount of the catalyst, and catalyst type (Scheme 2).

**Fig. 1:**
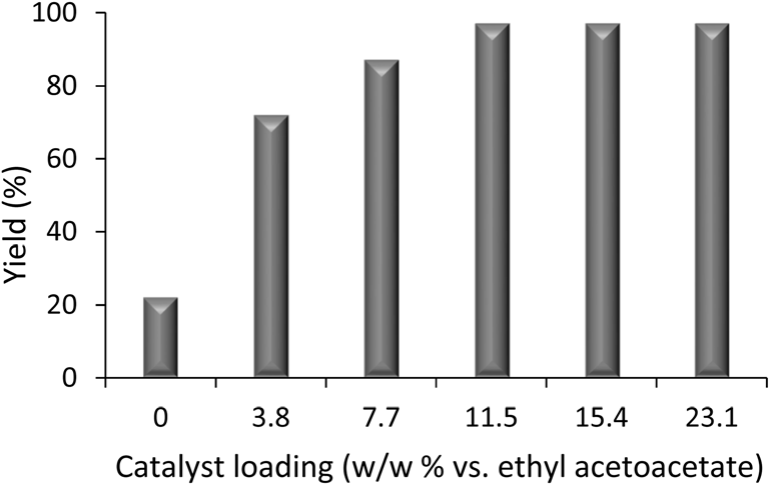
Effect of catalyst loading on the yield. Catalyst loading is expressed as weight percent of Fe_3_O_4_@GO-NH relative to ethyl acetoacetate.

In the first step, we optimized the amount of the catalyst to determine its effect on the reaction yield. As depicted in Fig. 1, the yield improves significantly as the catalyst loading increases from 0 to 11.5 wt%, based on ethyl acetoacetate. This indicates that the catalyst effectively promotes the reaction by providing active sites necessary for the reaction mechanism. Beyond 11.5 wt%, further increases in the catalyst loading (15.4 and 23.1 wt%) did not enhance the yield, suggesting that the reaction has reached a saturation point where the maximum catalytic efficiency is achieved.

**Fig. 2:**
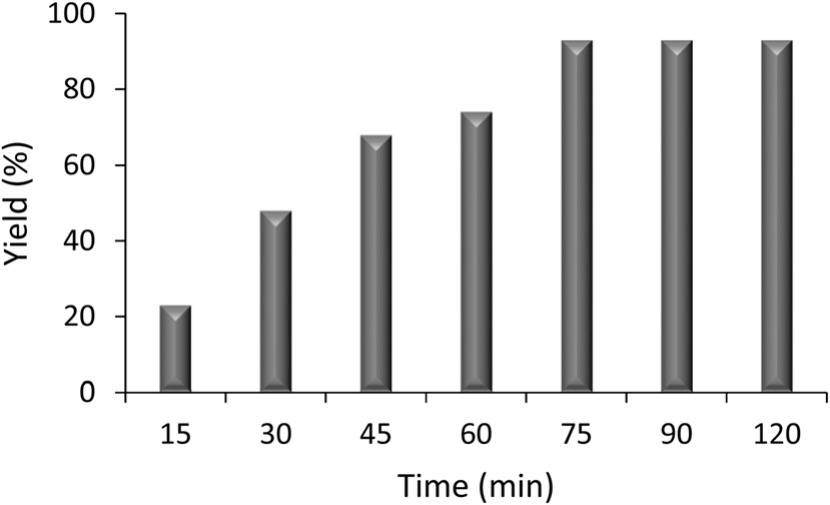
Effect of the reaction time on the yield.

Further optimization focused on the reaction time parameter (Fig. 2). The figure illustrates a steady increase in the yield as the reaction time progresses from 15 to 75 minutes. At 75 minutes, the yield reached its maximum value, while further extending the reaction time to 90 and 120 minutes did not result in any further increase in the yield. This suggests that 75 minutes is the optimal reaction time for maximizing yield in the reaction.

**Fig. 3:**
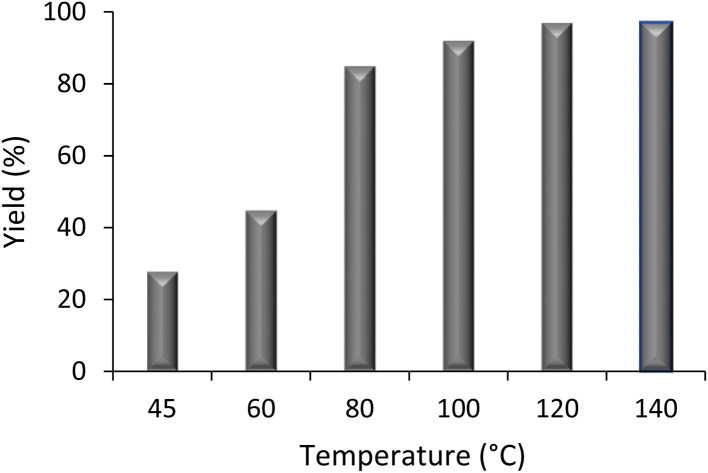
Effect of the reaction temperature on the yield.

Further investigation examined the effect of reaction temperature on the yield. Thus, six model reactions were conducted, each using 0.015 g Fe_3_O_4_@GO-NH (a catalyst loading of 11.5 wt%) for 75 minutes at temperatures between 45 °C and 140 °C (Fig. 3). The data show that 130 °C is the optimal reaction temperature, and the yield rises steadily from approximately 20% at 45 °C to 97% at 130 °C, reflecting the significant effect of temperature on the Biginelli product yield. However, higher temperatures, such as 140 °C, do not significantly improve yield. As a result, the optimized reaction conditions were determined to include a reaction time of 75 minutes, a temperature of 130 °C, and the catalyst loading of 11.5 wt%.

### 3.3. Efficiency of Fe_3_O_4_/GO-NH

We then evaluated several related catalysts in the model reaction under the optimized reaction conditions to compare their efficiency with that of Fe_3_O_4_@GO-NH. As shown in Table 1, Fe_3_O_4_@GO-NH provided the highest yield among the tested catalysts while offering ease of recyclability. Its superiority over Fe_3_O_4_@rGO-NH (rGO is reduced graphene oxide) stems from GO's higher density of –COOH groups, which adds a more Brønsted acidic sites together with the piperazine basic sites. These cooperative acid–base surface functionalities improve the reaction yield.

**Table 1:** Comparison of the catalytic efficiency of Fe_3_O_4_@GO-NH with various related catalysts in the model Biginelli reaction^a^

Entry	Catalyst	Yield (%)	Ref.
1	GO	52	This work
2	GO-NH	58	This work
3	Fe_3_O_4_	38	This work
4	Fe_3_O_4_	45	13
5	Fe_3_O_4_@rGO-NH	63	This work
6	Fe_3_O_4_@GO-NH	97	This work

a Reaction conditions: benzaldehyde (1 mmol), urea (1.3 mmol), ethyl acetoacetate (1 mmol), solvent-free, catalyst loading (0.015 g, 11.5 wt%), 130 °C, 75 min.

Like Fe_3_O_4_@GO-NH, the PZC value was determined for Fe_3_O_4_@rGO-NH to demonstrate the expected decrease in the –COOH content of Fe_3_O_4_@rGO-NH by the reduction. A value of 5.21 for Fe_3_O_4_@rGO-NH was obtained (Fig. S9 in SI), confirming its decreased catalytic activity relative to Fe_3_O_4_@GO-NH due to less Brønsted acidic sites for the catalysis.

#### 3.3.1. Synthesis of other Biginelli products

**Scheme 3:**
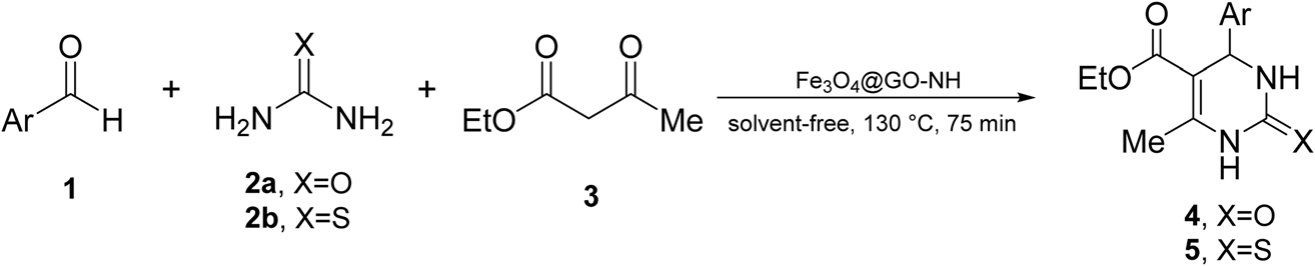
Solvent-free Biginelli reaction catalyzed by Fe_3_O_4_@GO-NH.

Next, the scope of our method for synthesizing various Biginelli products was investigated by applying the optimized conditions to different substrates, as shown in Scheme 3 and Table 2. The yield data demonstrate that, without requiring significant modifications to the protocol, our approach is highly effective across a wide range of substrates. This versatility highlights the robustness and efficiency of our method for the Biginelli reaction.

**Table 2:** Aldehyde starting materials and yield values of the synthesized Biginelli products^a^

Entry	ArCHO	X	Biginelli product	Yield (%)	mp (°C)	
					Found (from ethanol)	Reported (lit. reference)
1	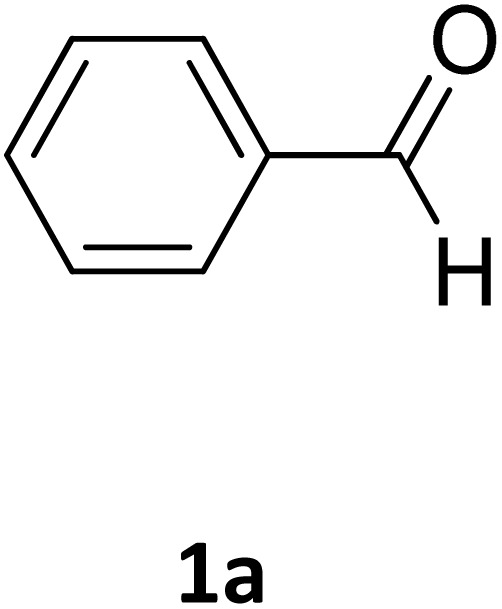	O	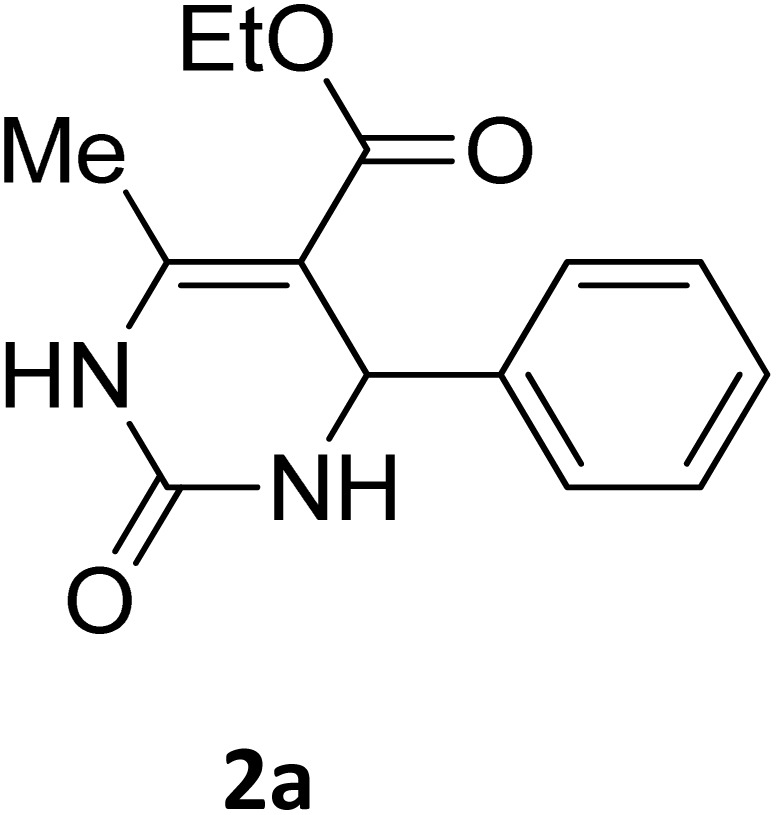	97	207	210 (ref. 14)
2	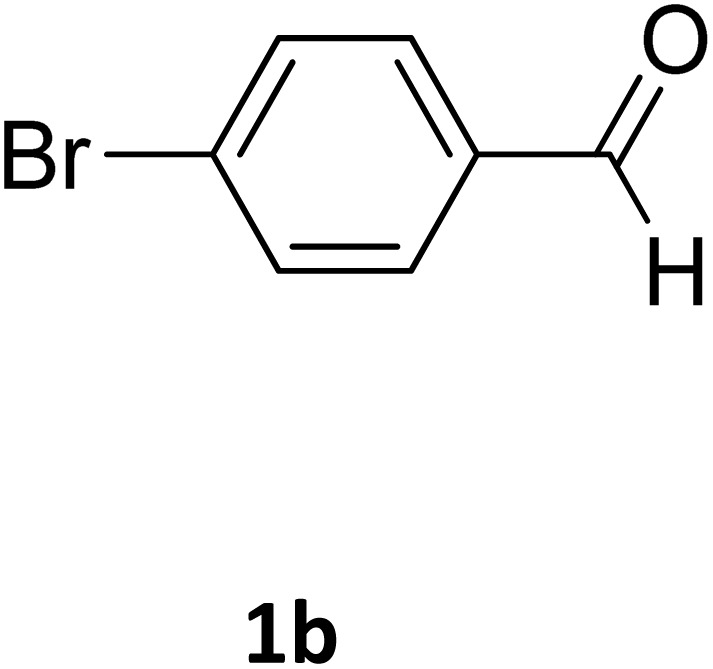	O	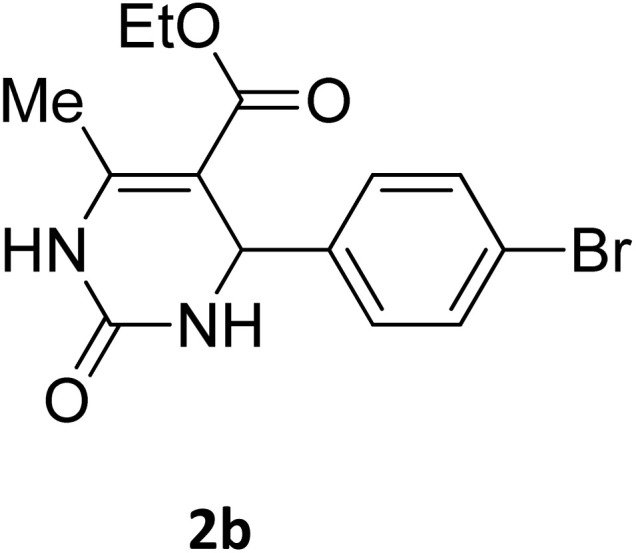	98	215	215 (ref. 15)
3	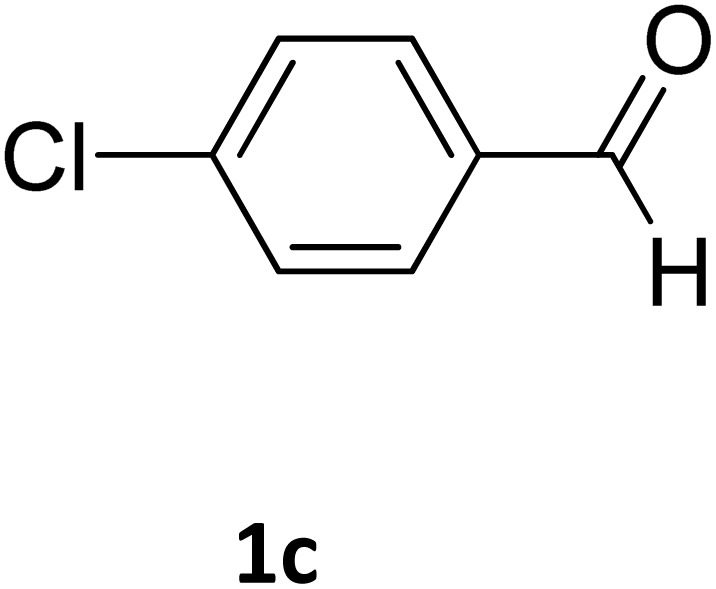	O	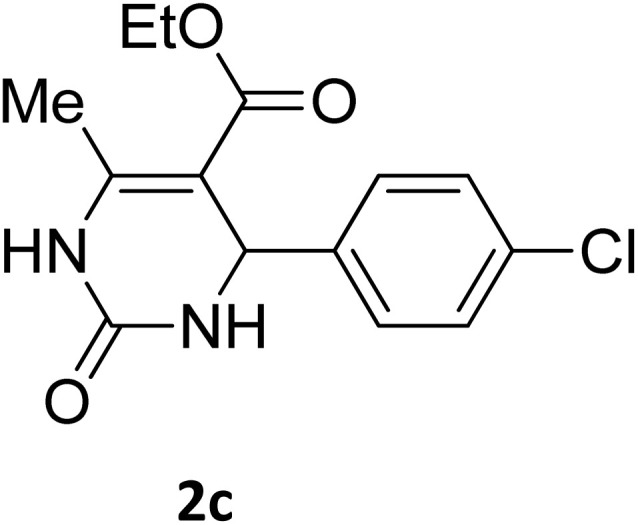	97	210	212 (ref. 16)
4	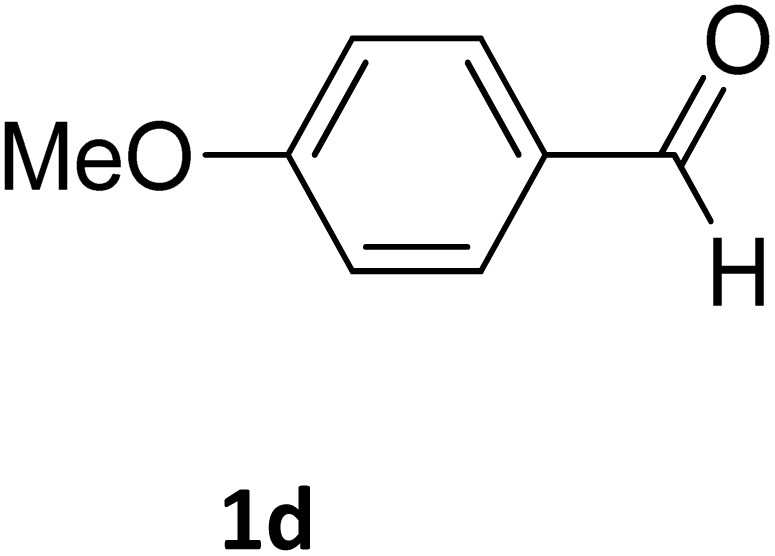	O	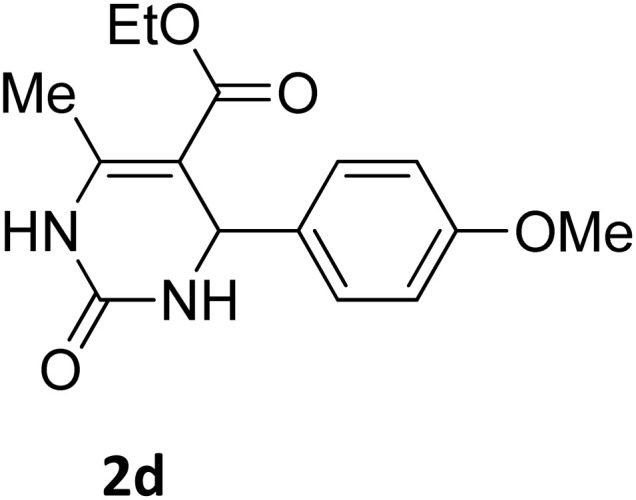	93	202	203 (ref. 17)
5	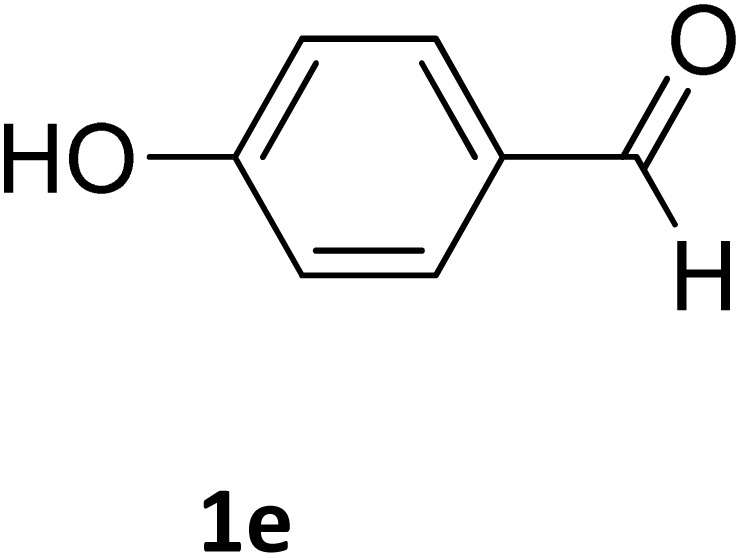	O	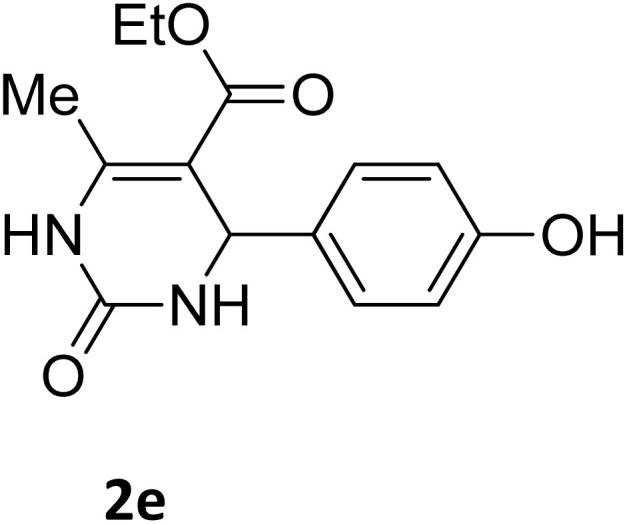	94	204	204 (ref. 18)
6	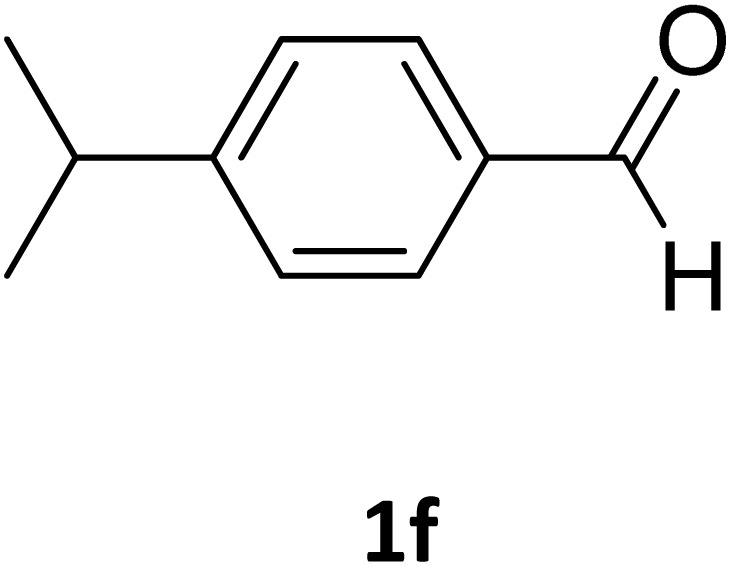	O	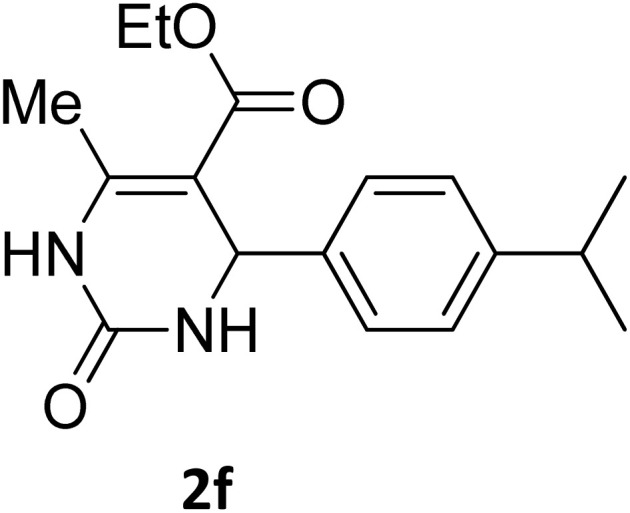	85	198	197 (ref. 19)
7	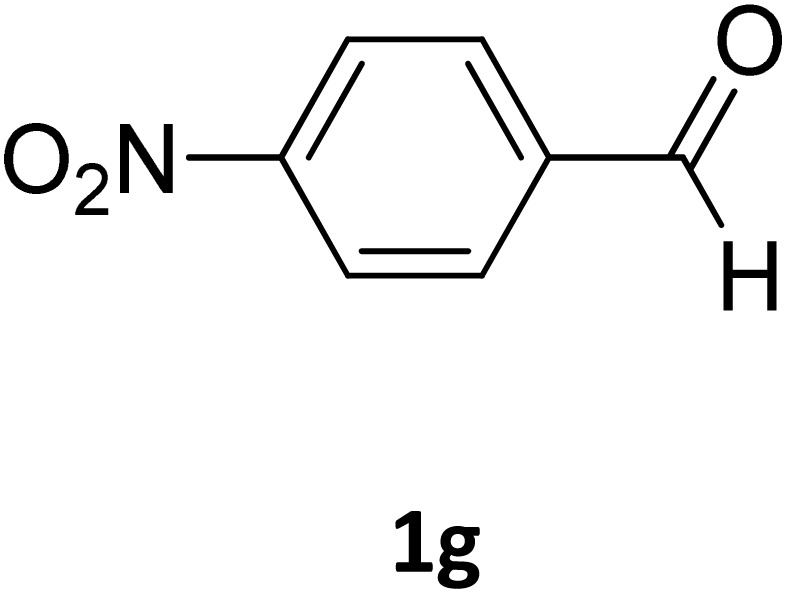	O	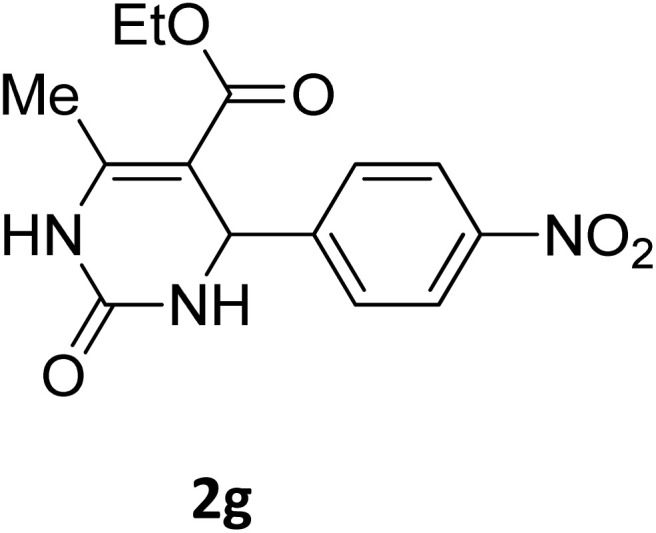	98	208	210 (ref. 20)
8	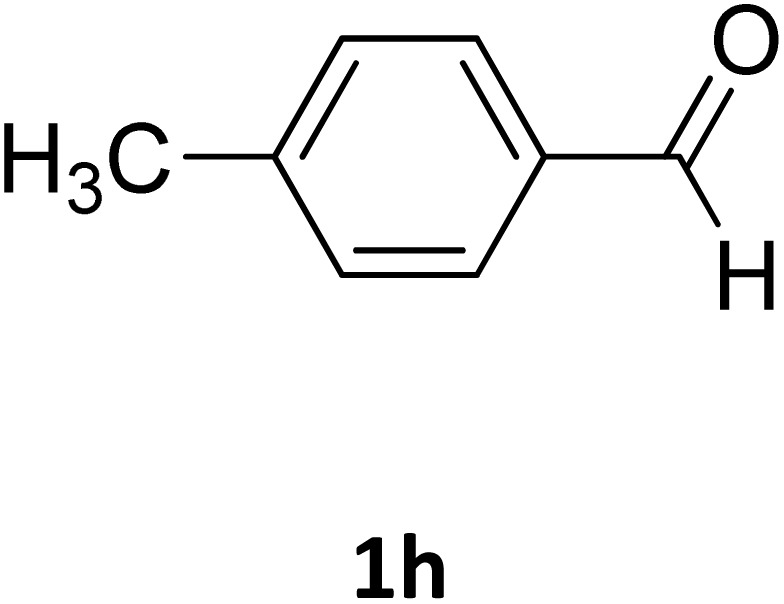	O	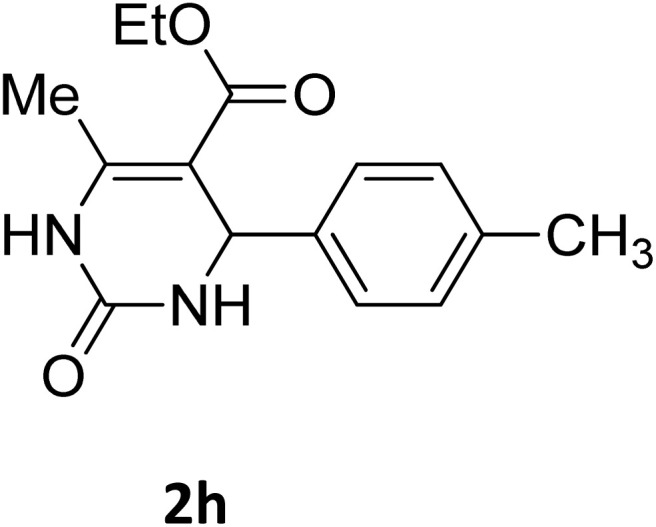	92	209	207 (ref. 21)
9	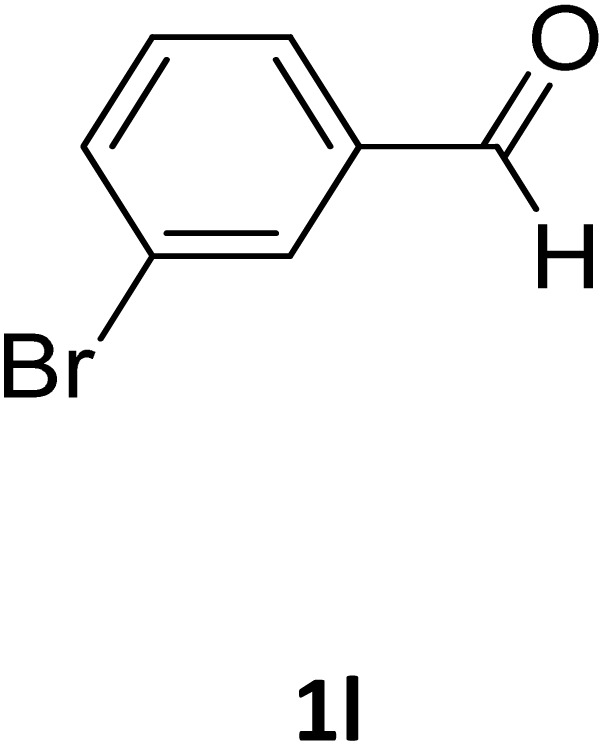	O	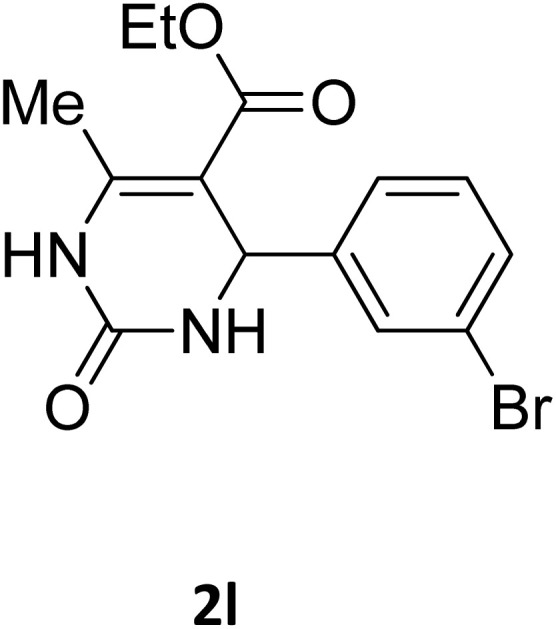	95	200	198 (ref. 22)
10	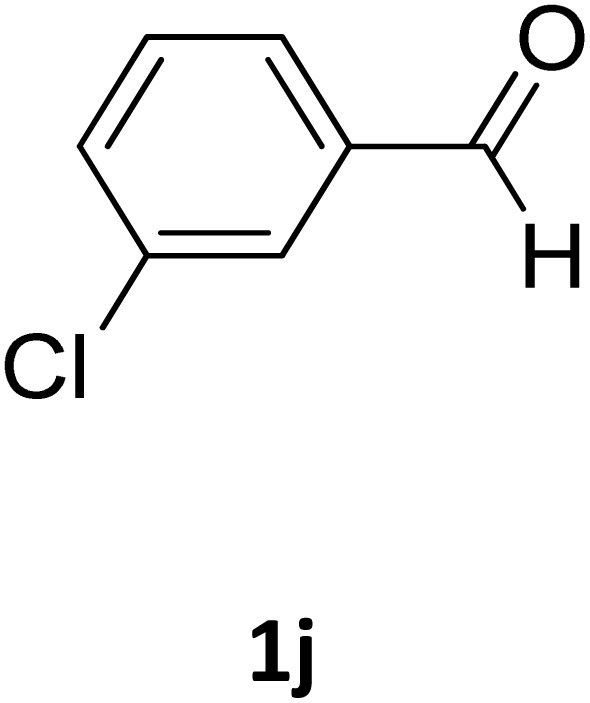	O	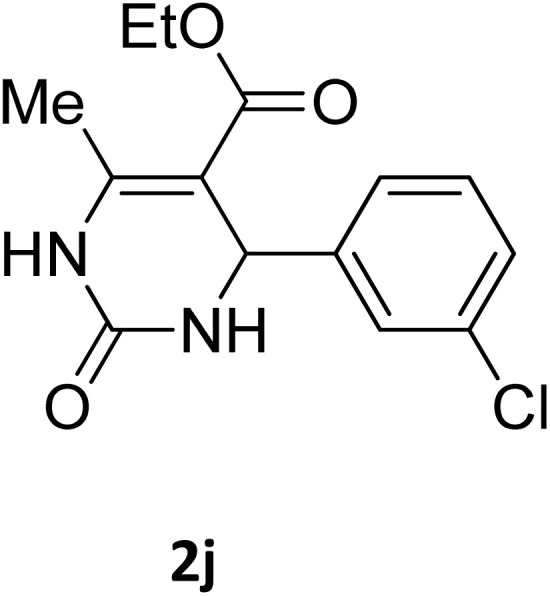	97	201	198 (ref. 23)
11	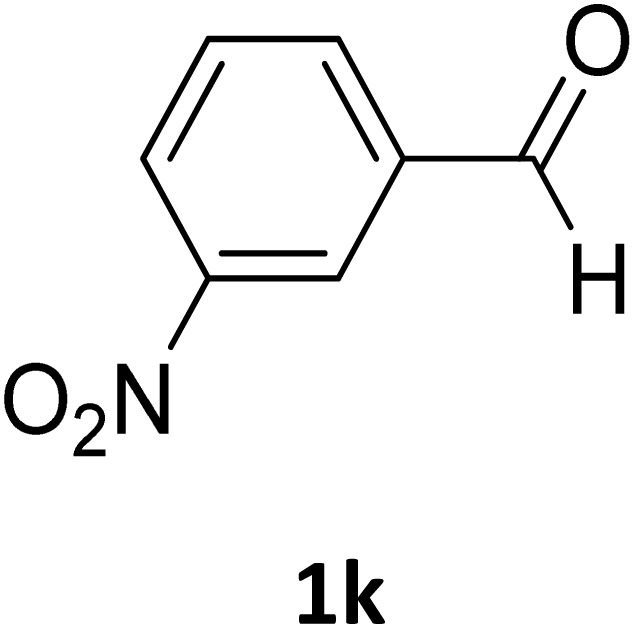	O	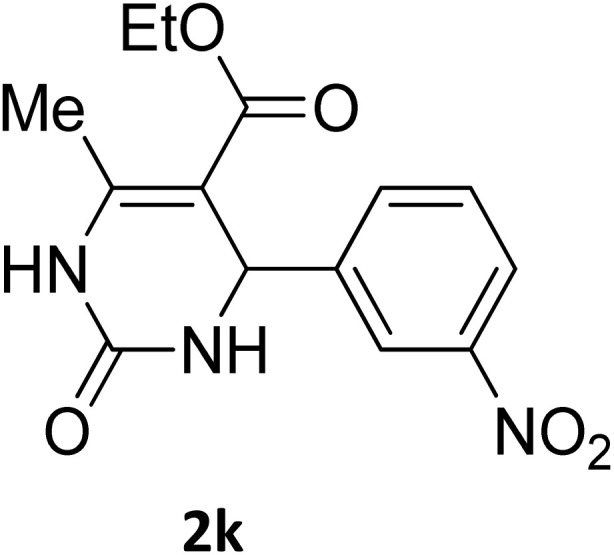	95	210	210 (ref. 24)
12	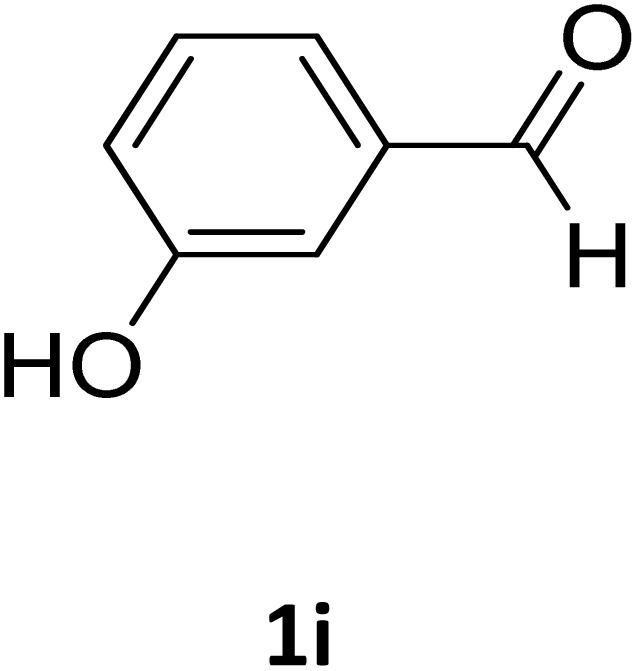	O	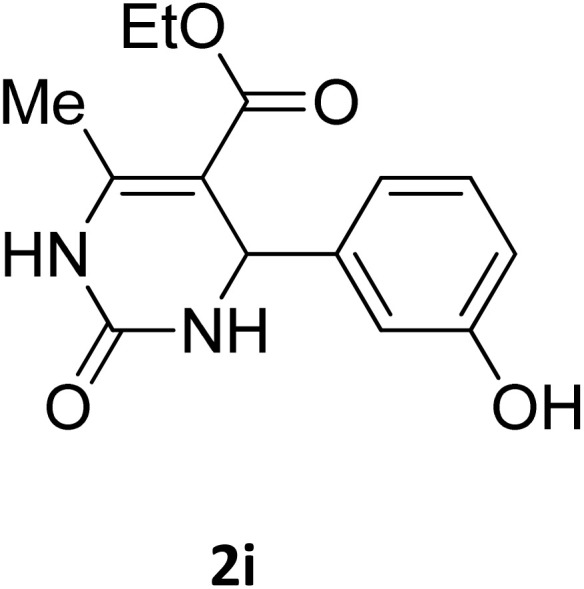	73	210	212 (ref. 25)
13	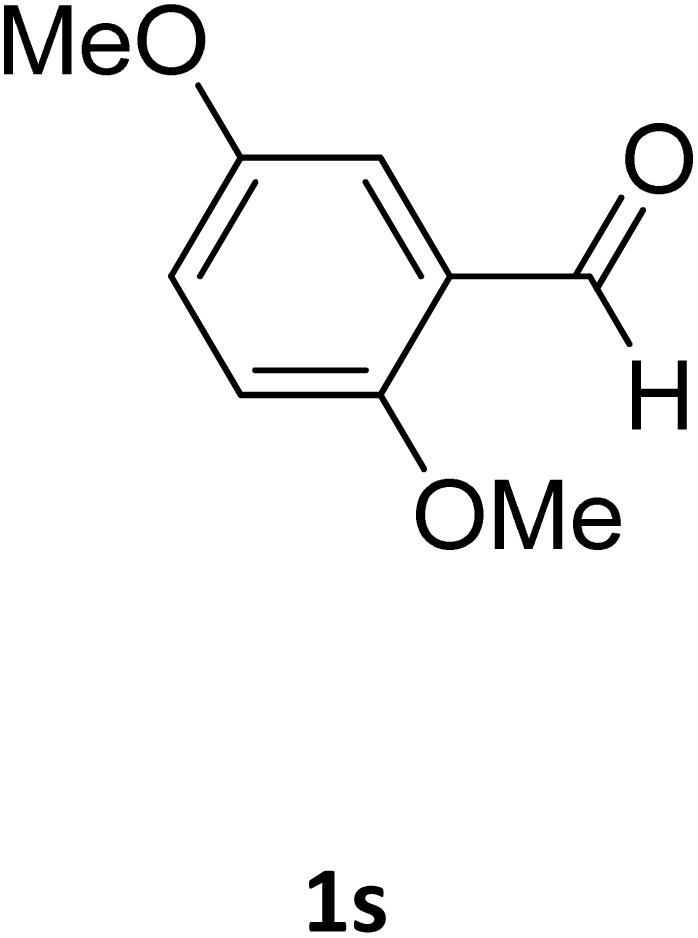	O	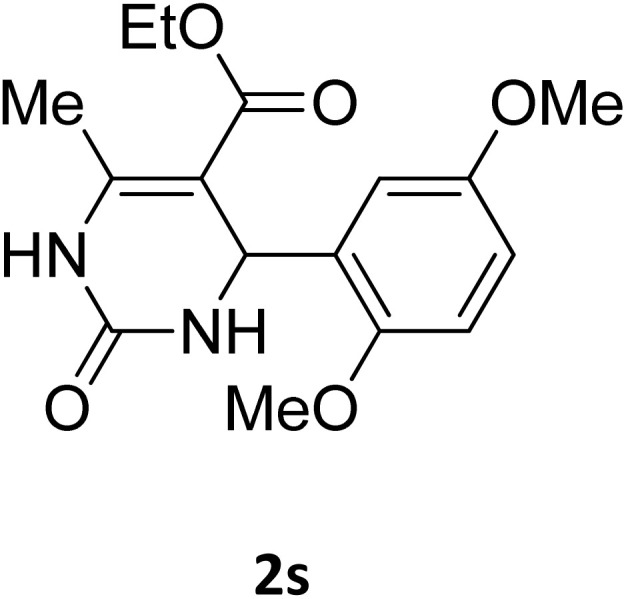	97	189	188 (ref. 26)
14	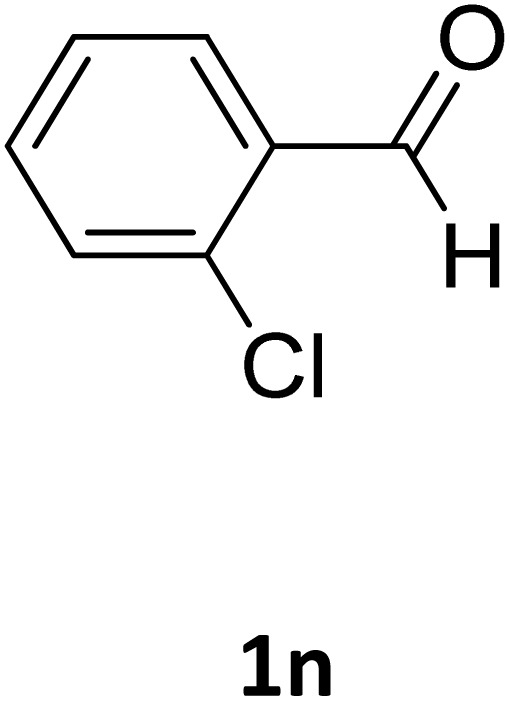	O	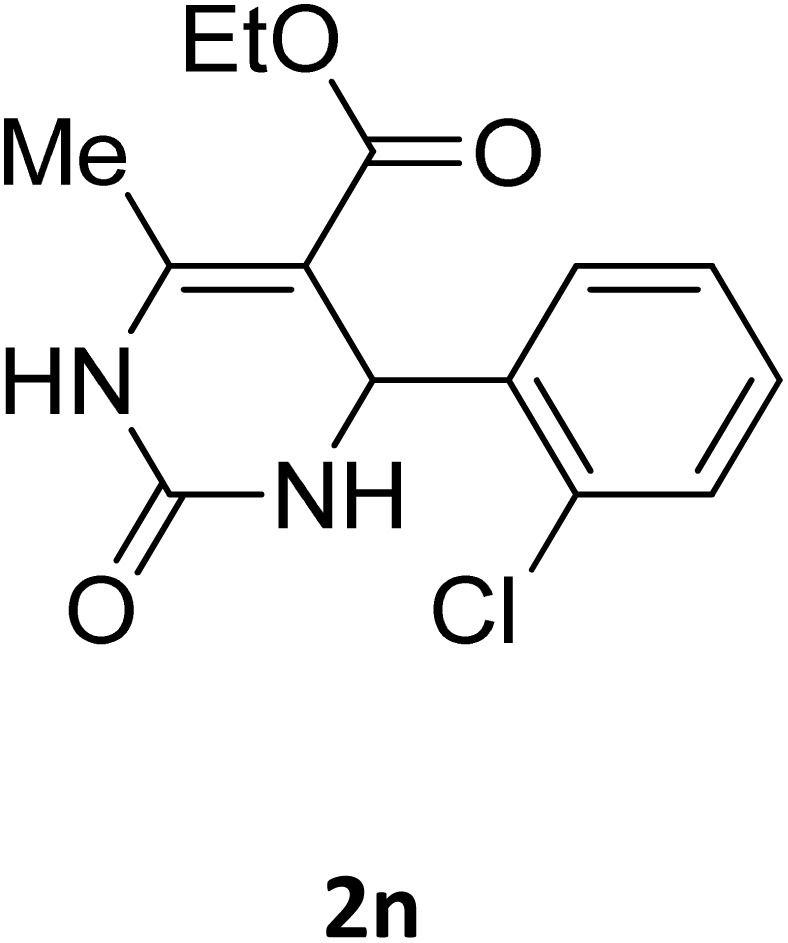	94	220	221 (ref. 27)
15	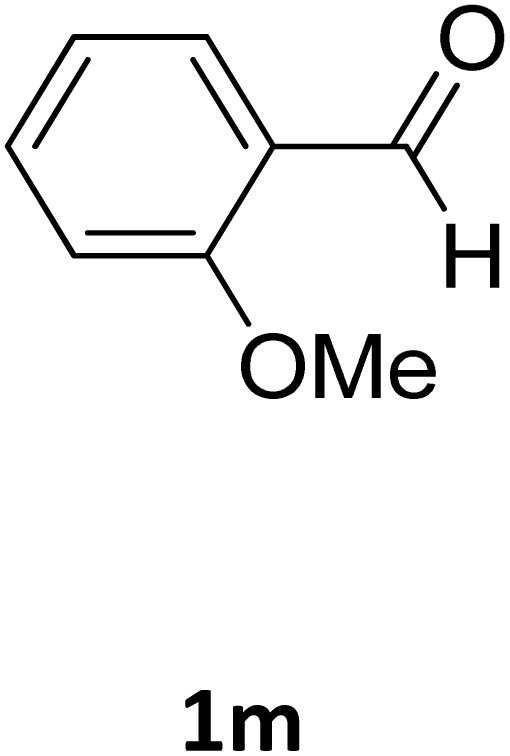	O	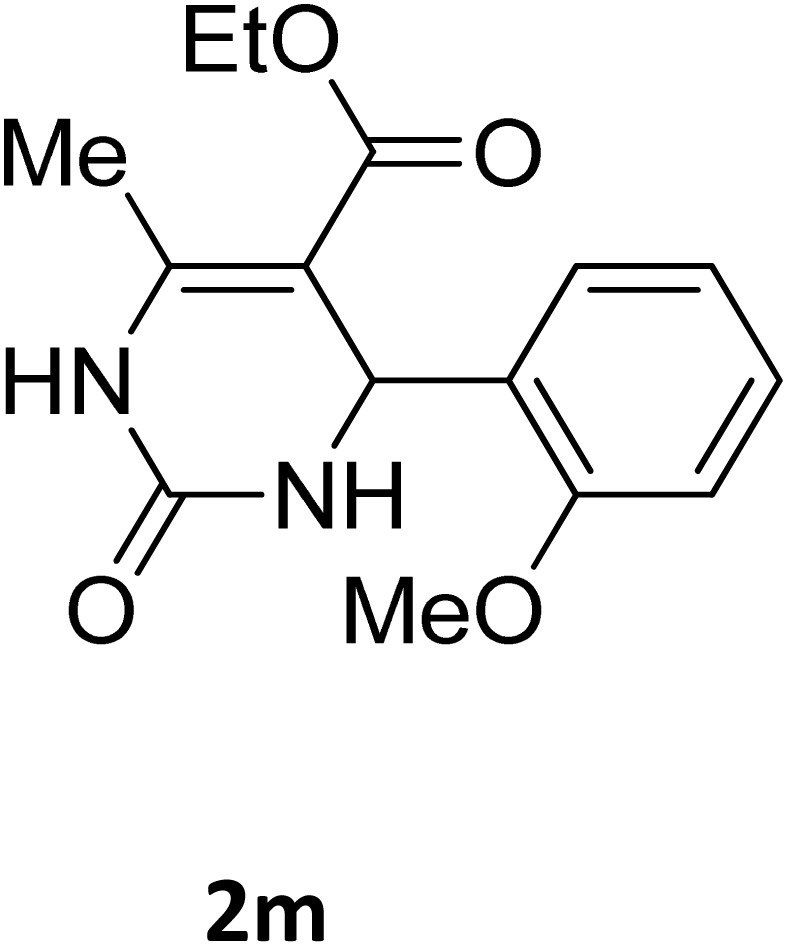	72	202	202 (ref. 28)
16	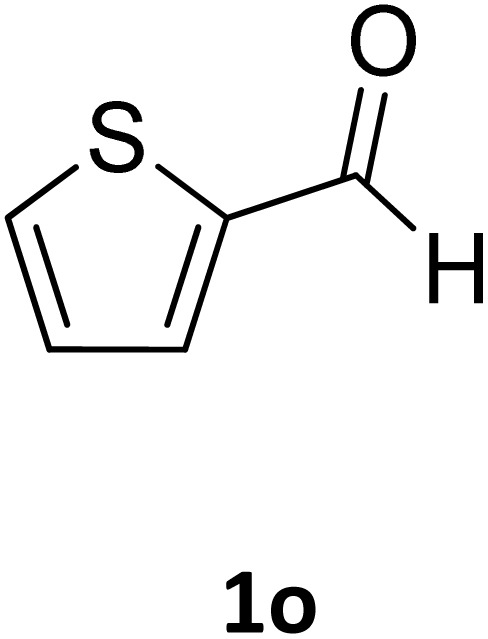	O	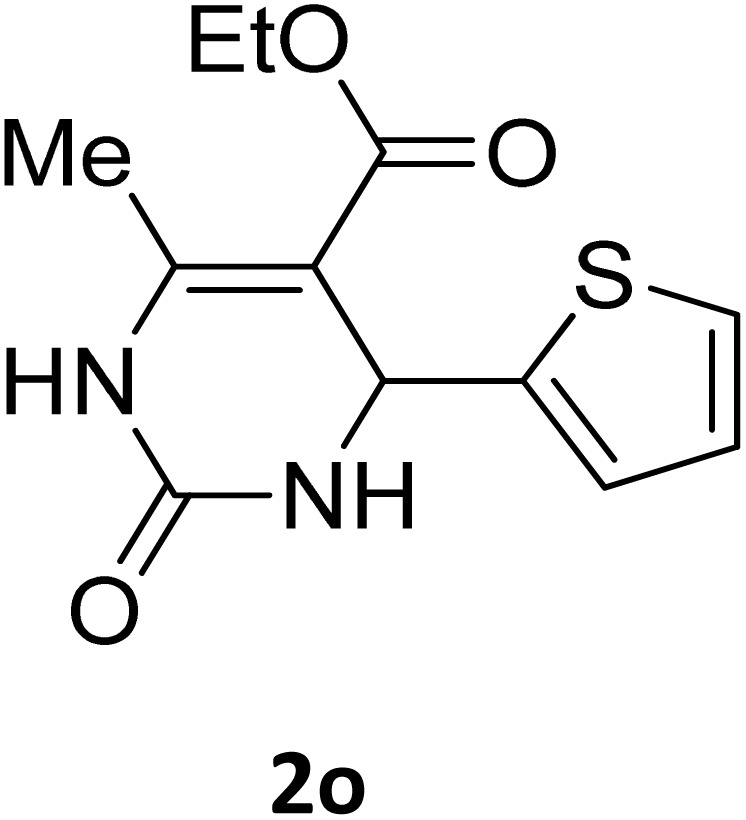	91	197	197 (ref. 29)
17	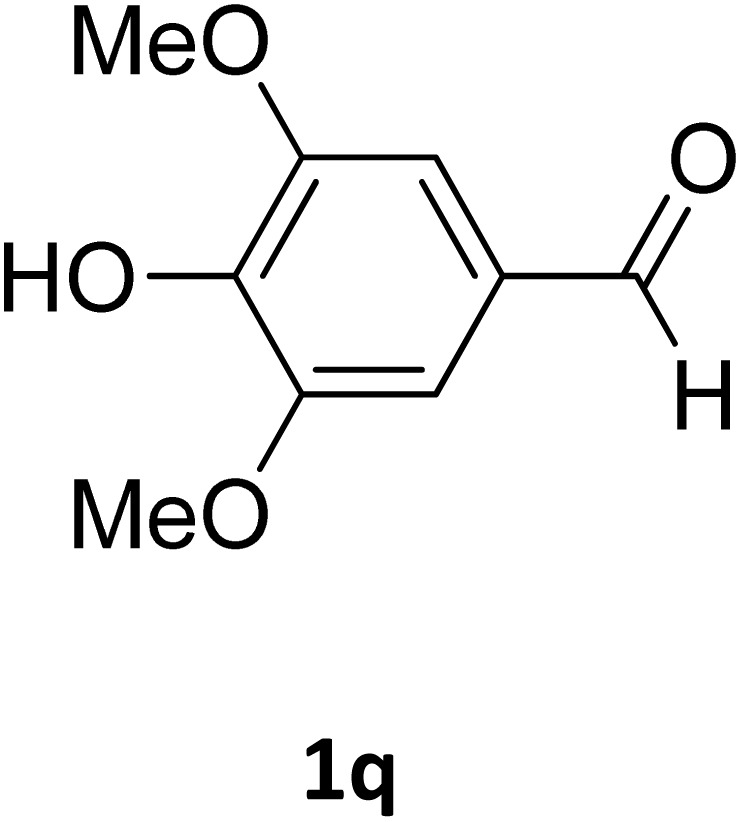	O	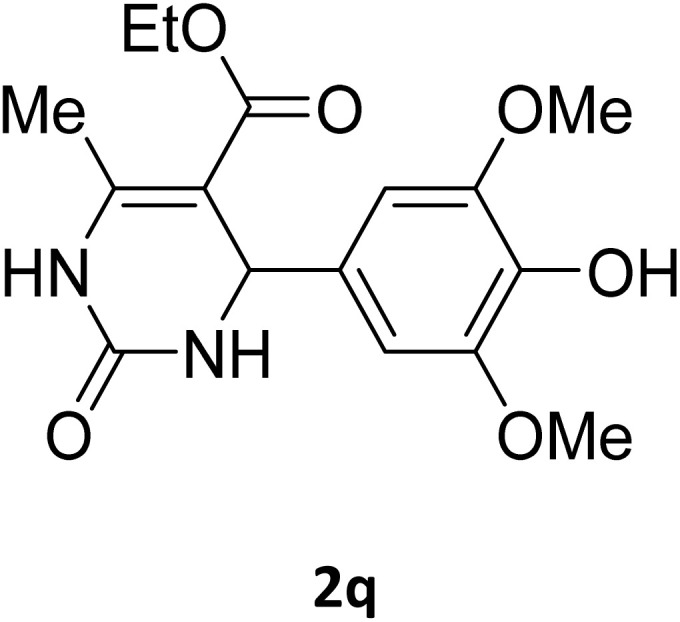	75	201	183 (ref. 30)
18	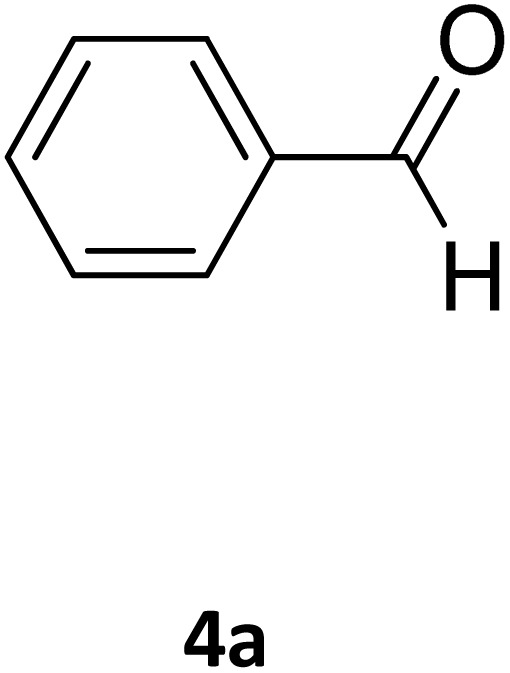	S	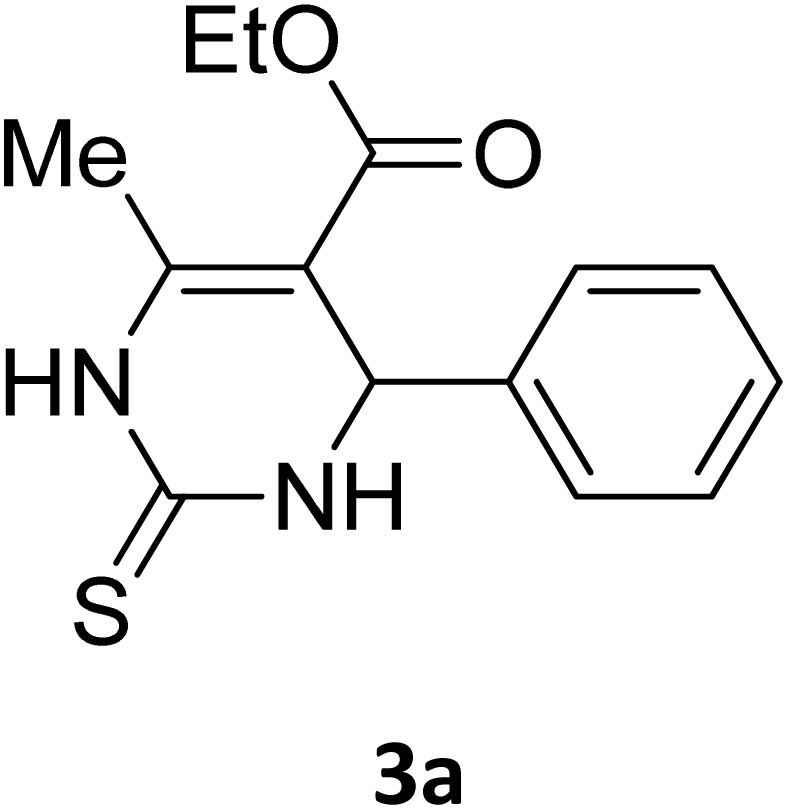	98	190	190 (ref. 31)
19	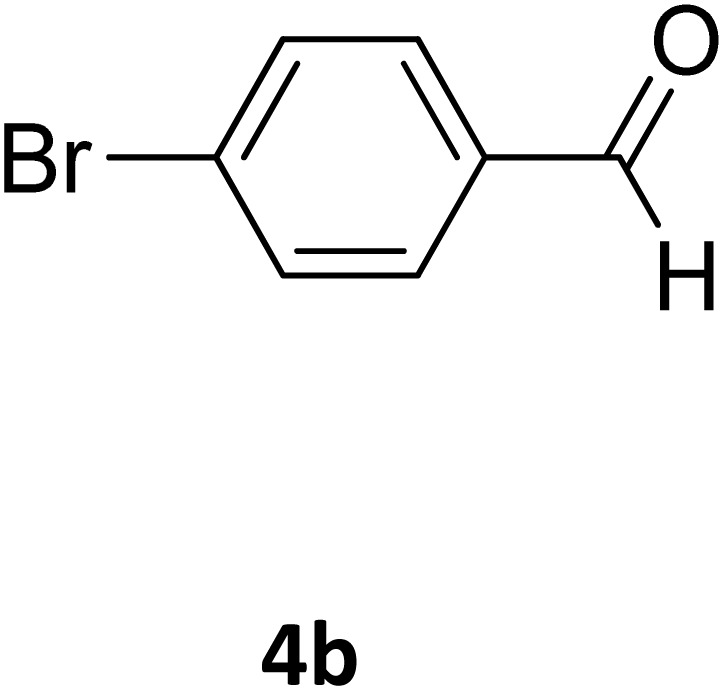	S	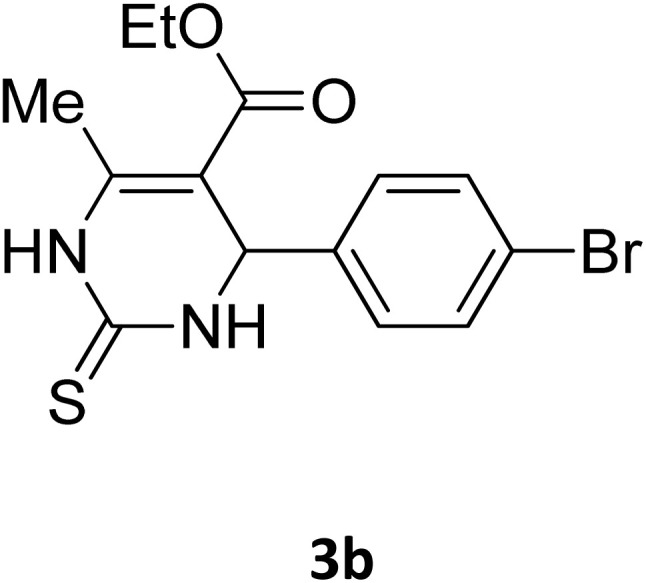	96	180	180 (ref. 32)
20	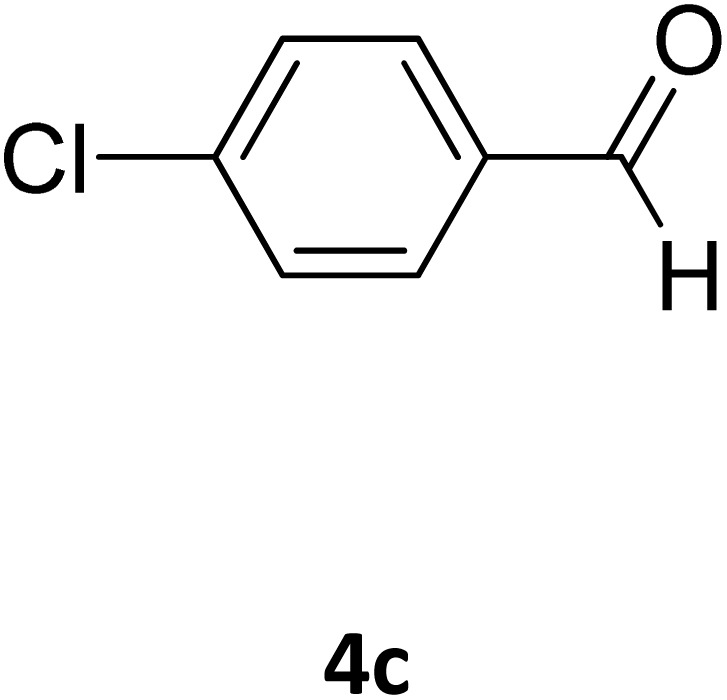	S	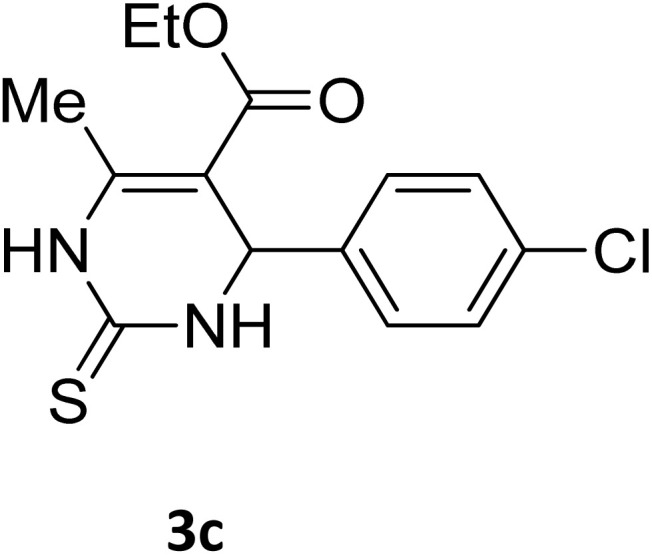	96	183	181 (ref. 33)
21	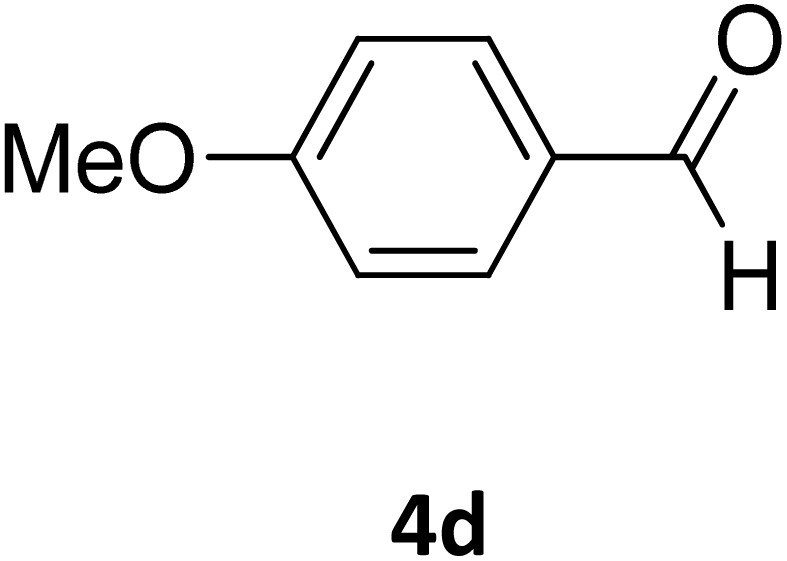	S	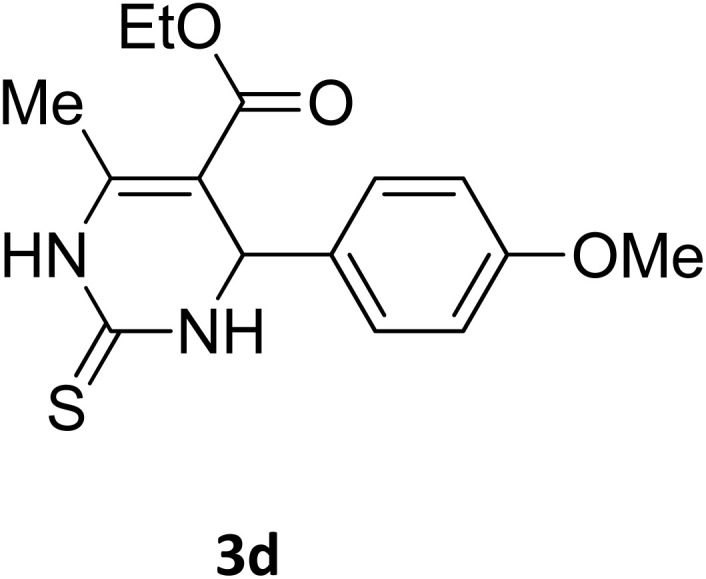	73	202	207 (ref. 34)
22	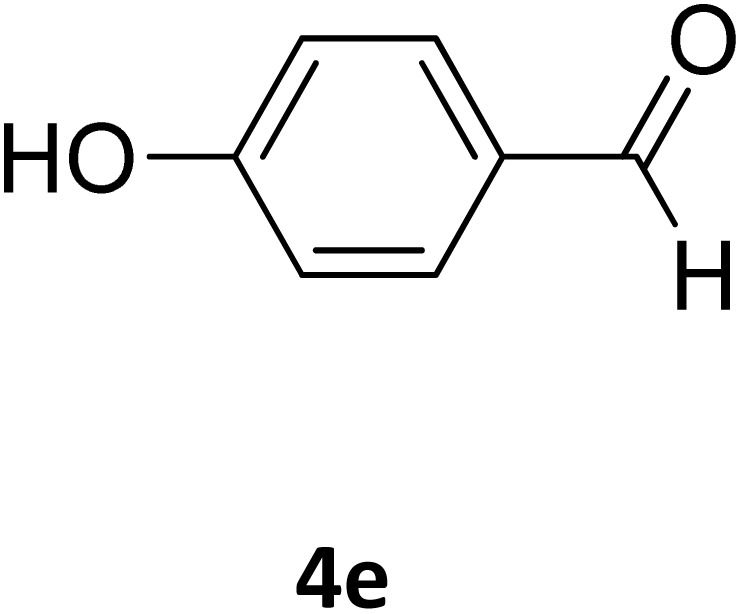	S	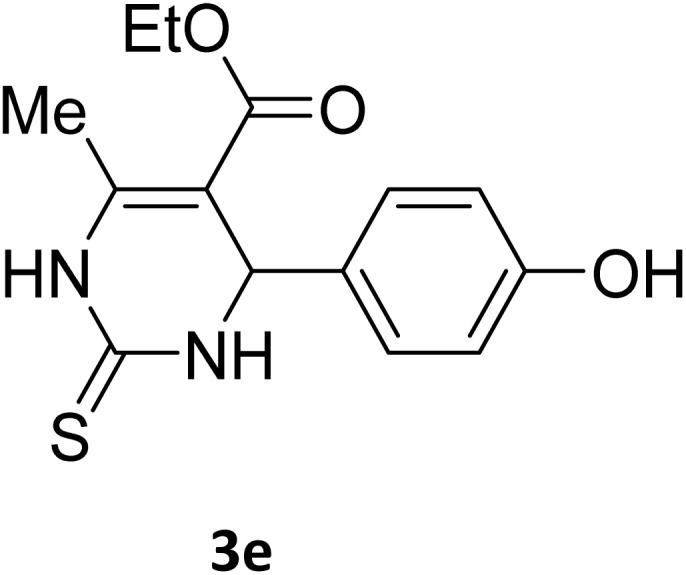	58	180	185 (ref. 35)
23	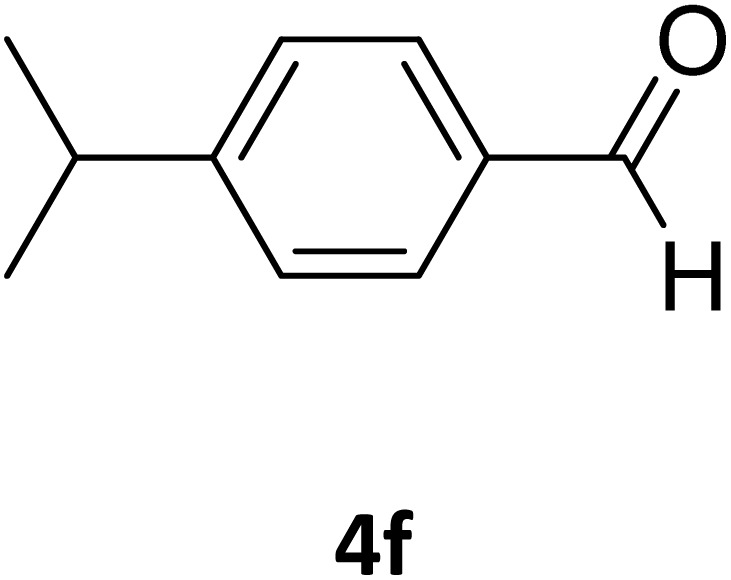	S	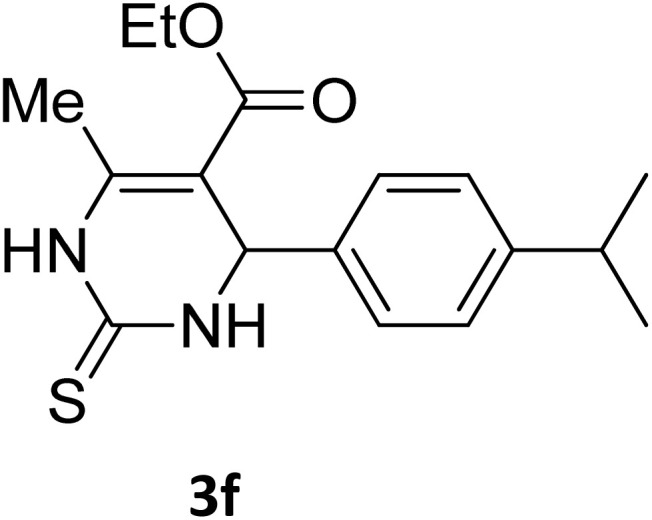	85	181	180 (ref. 36)
24	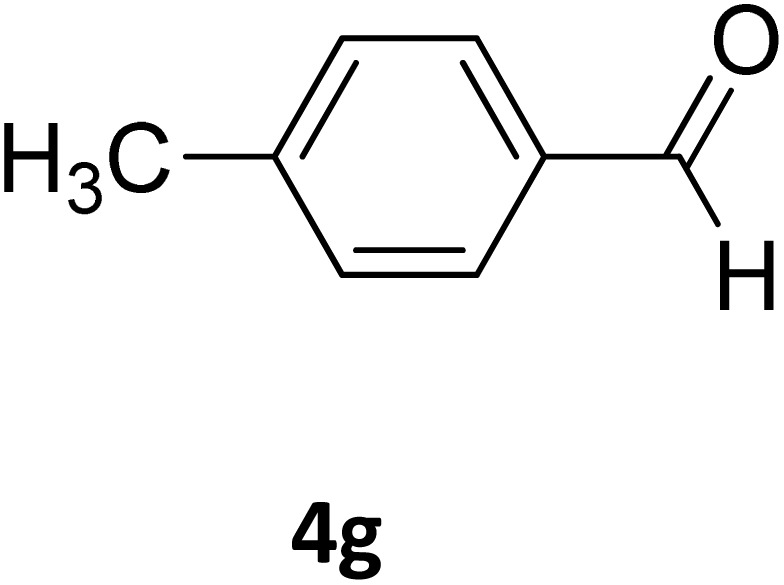	S	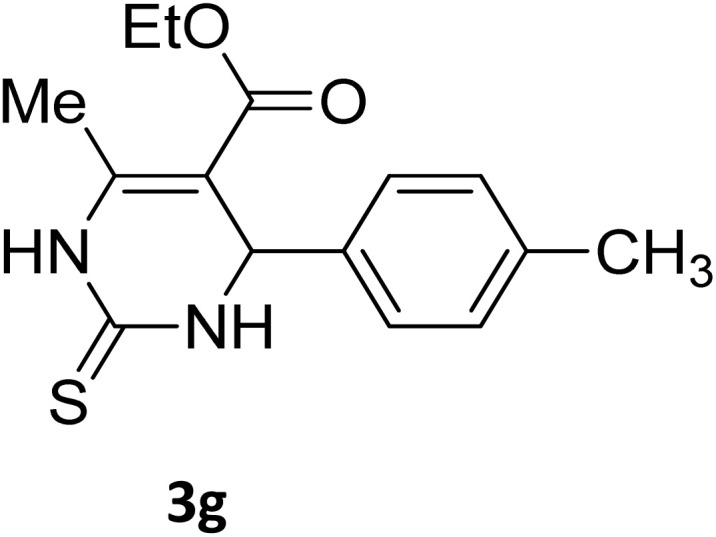	82	194	194 (ref. 37)
25	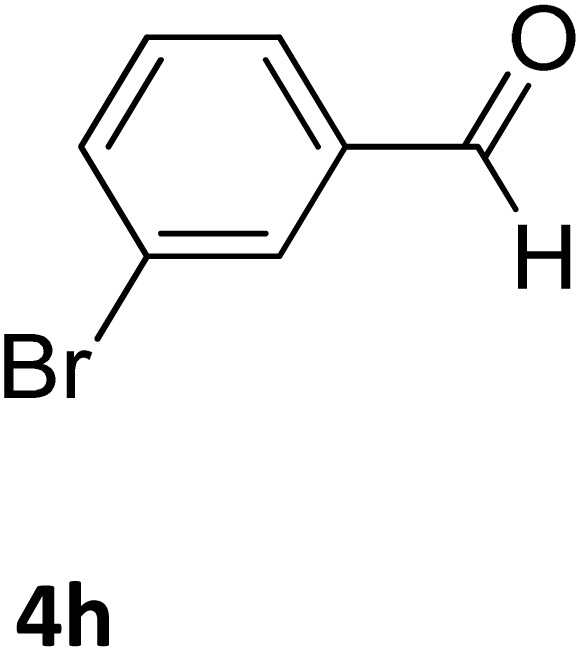	S	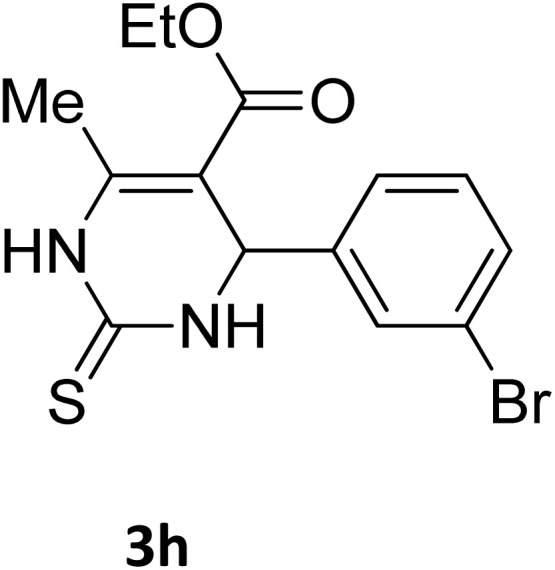	96	183	183 (ref. 38)
26	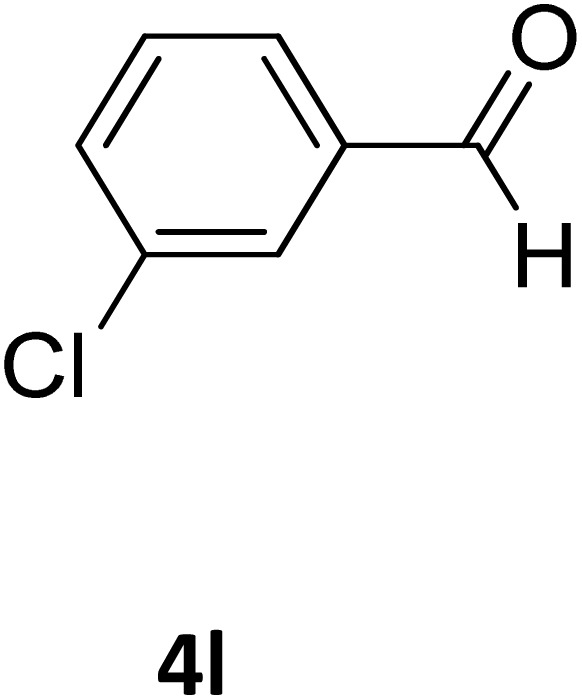	S	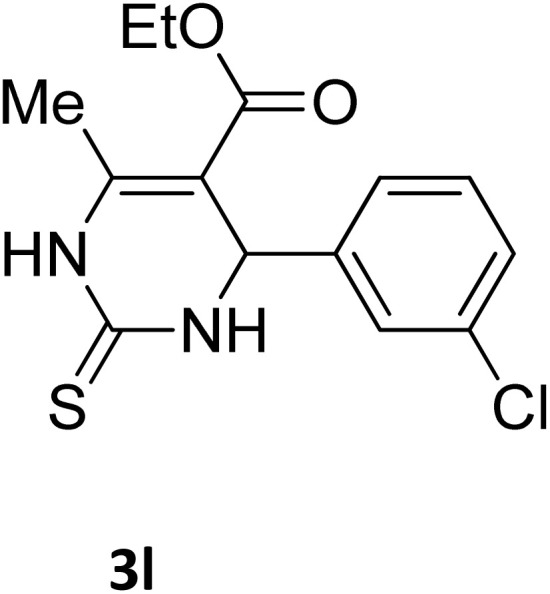	95	193	194 (ref. 39)
27	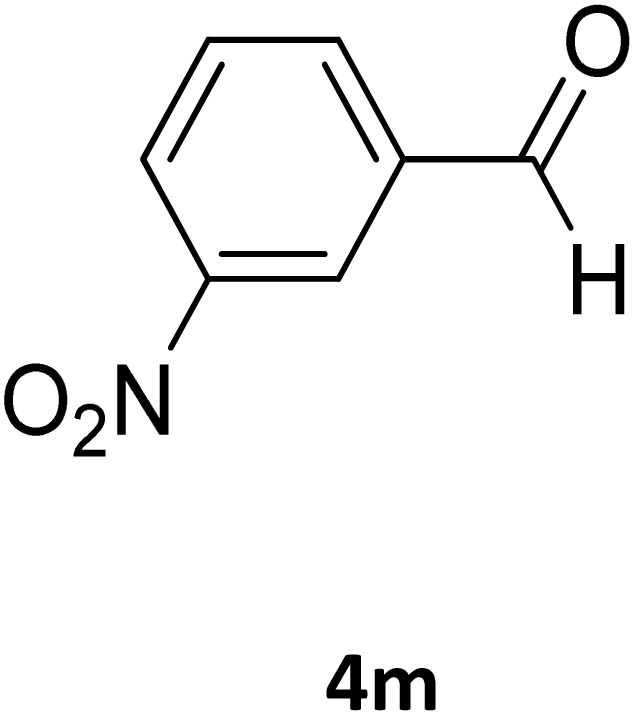	S	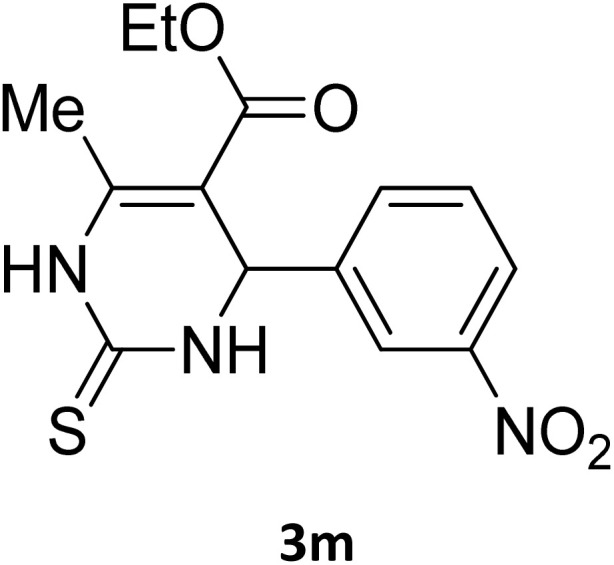	93	198	198 (ref. 40)
28	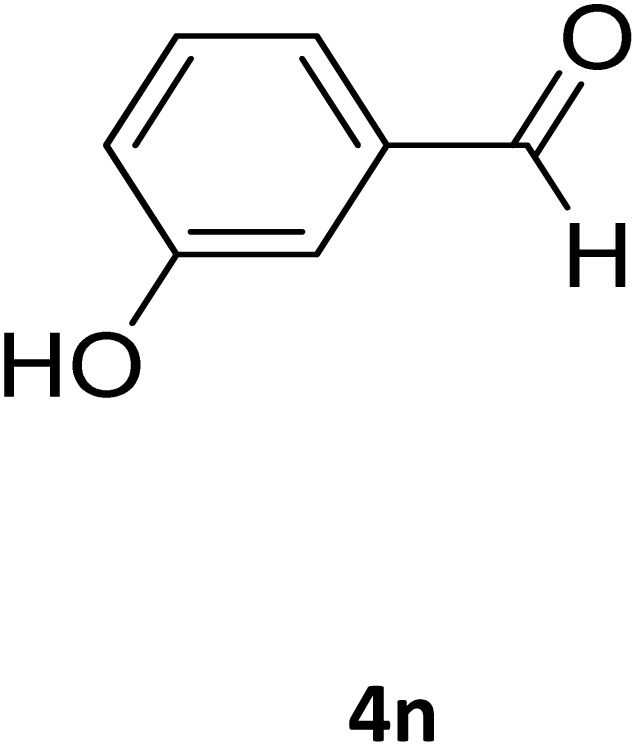	S	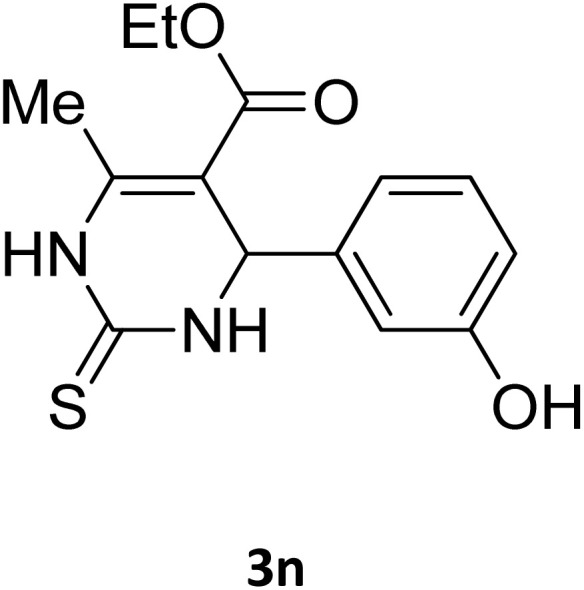	78	175	176 (ref. 41)
29	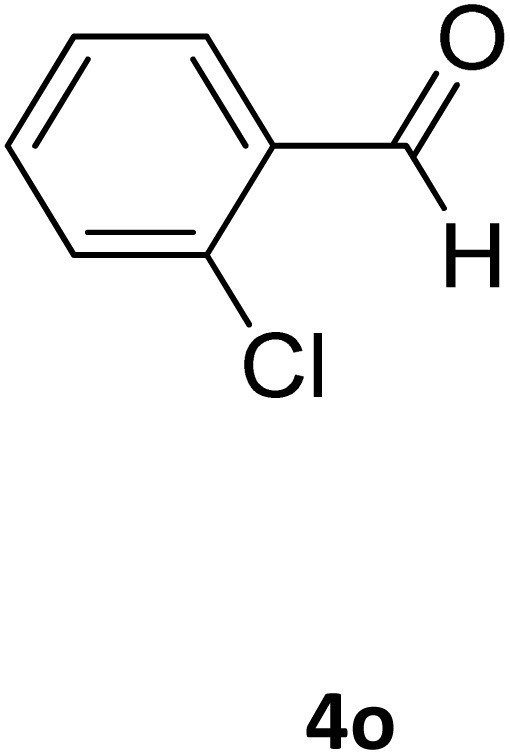	S	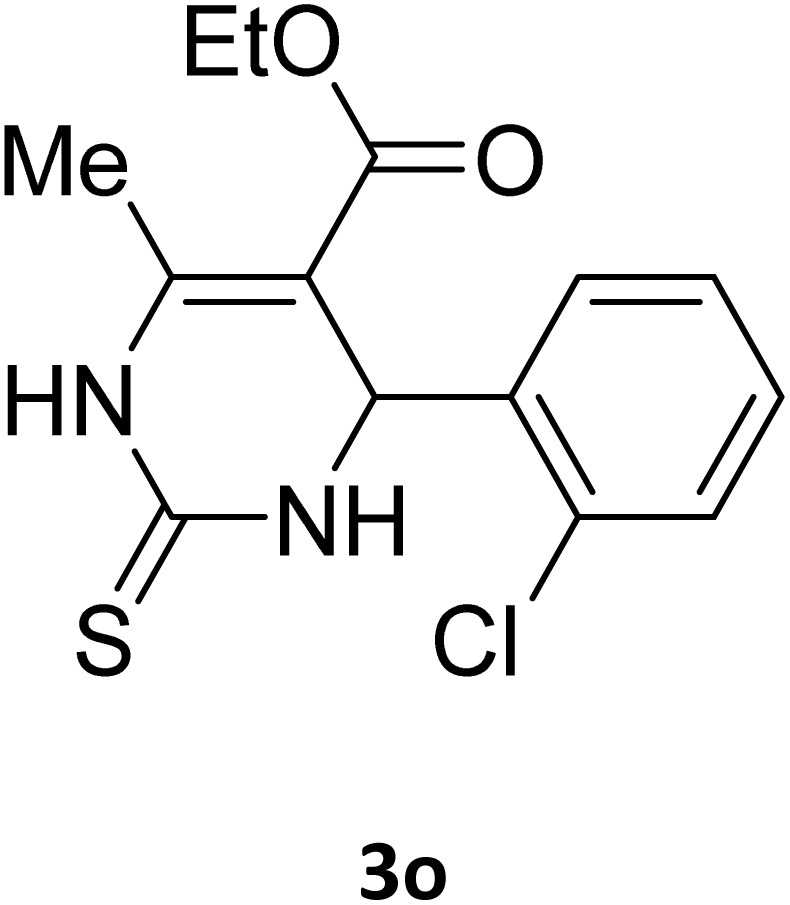	96	170	170 (ref. 42)
30	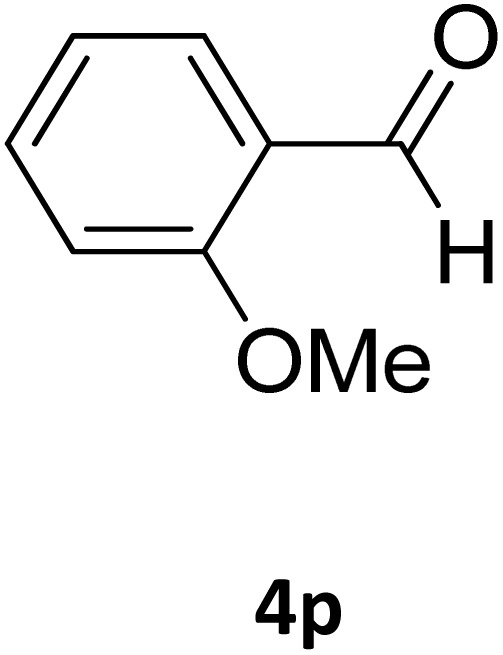	S	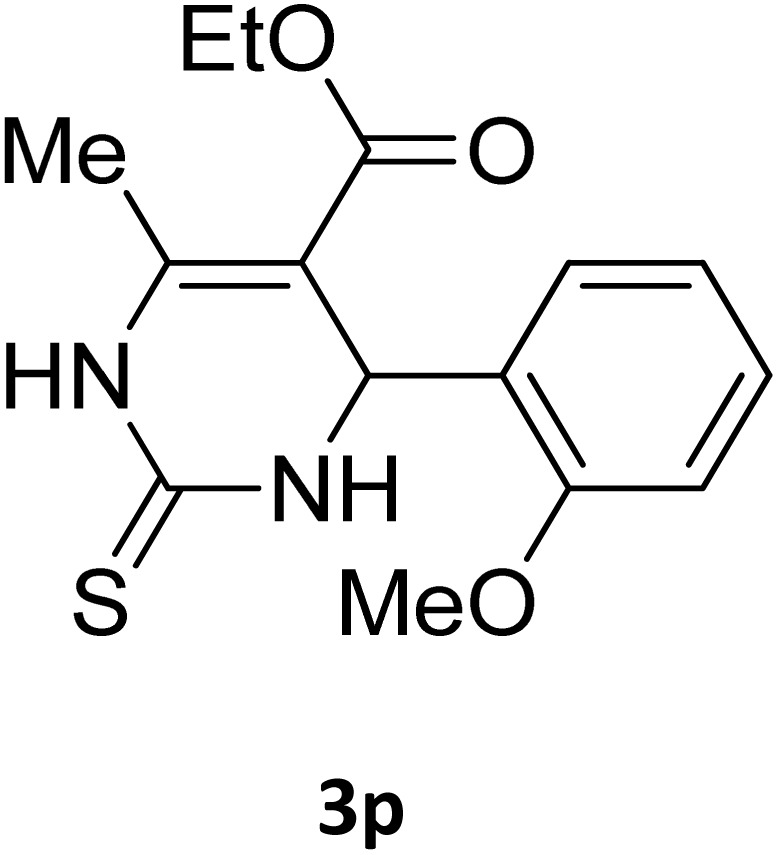	88	186	187 (ref. 43)
31	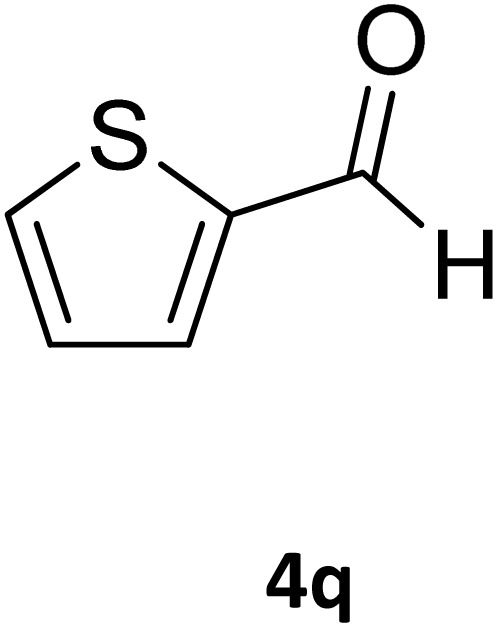	S	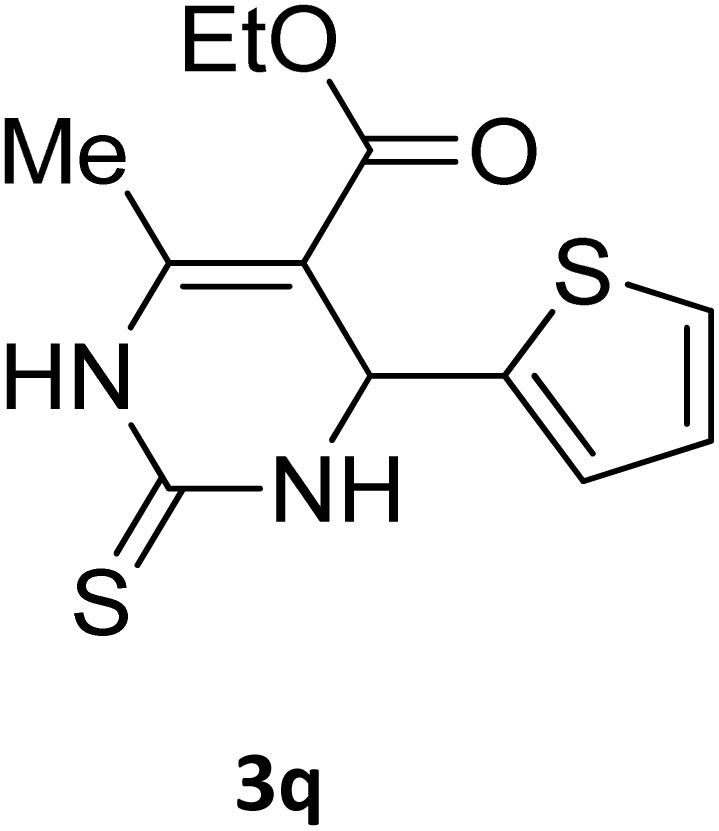	68	170	171 (ref. 44)
32	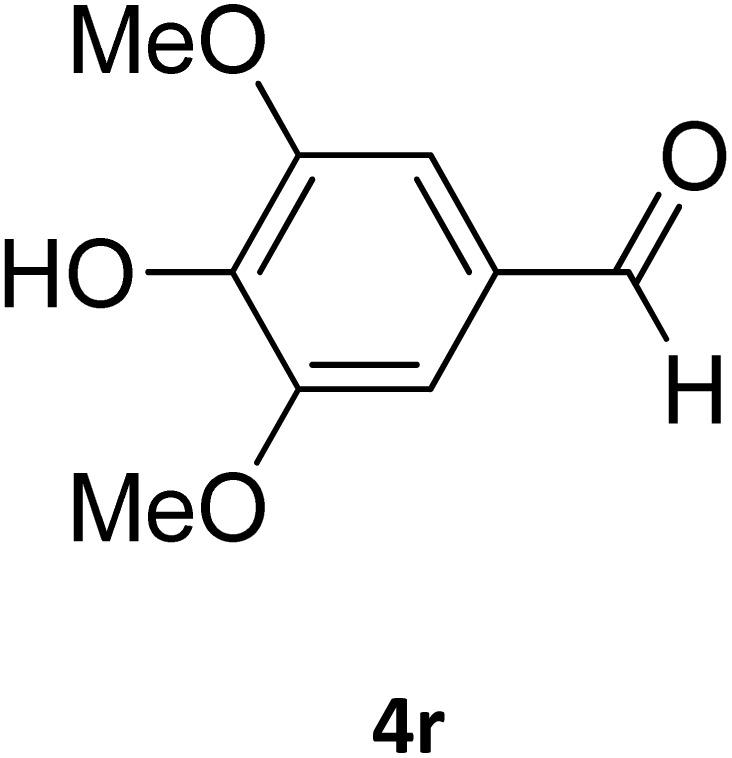	S	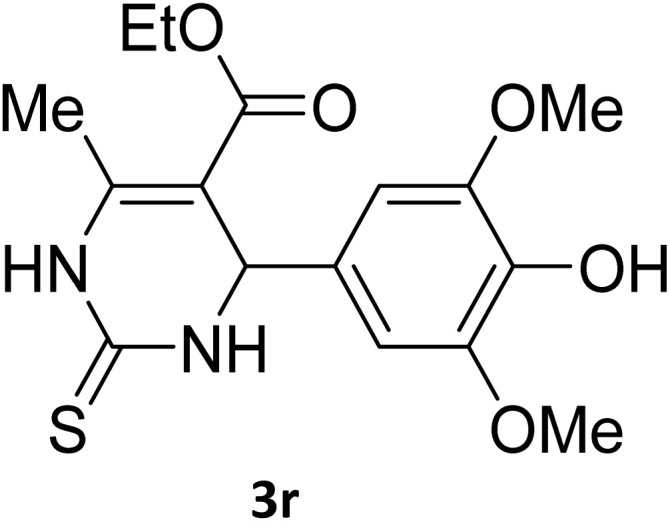	77	190	191 (ref. 41)

a Reaction conditions: starting aldehyde compound (1 mmol); urea (1.3 mmol); ethyl acetoacetate (1 mmol); solvent-free; Fe_3_O_4_@GO-NH loading (0.015 g, 11.5 wt%); time = 75 min; temperature = 130 °C.

#### 3.3.2. Reusing of catalyst (cyclic test)

**Fig. 4:**
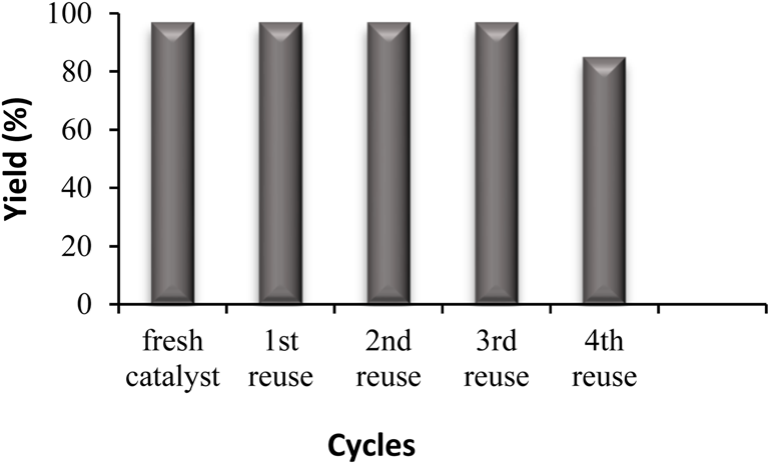
Reusability of Fe_3_O_4_@GO-NH.

Finally, the recovery and reusability of this heterogeneous catalyst were investigated as crucial aspects of its efficiency. To recover Fe_3_O_4_@GO-NH, ethanol (10 mL) was added to the residue after completion of the reaction (75 min). The mixture was refluxed for 30 min, cooled to room temperature, and the magnetic catalyst was easily collected using a magnet. The supernatant was decanted, leaving the catalyst attached to the magnet. The catalyst was then washed with ethanol (3 × 5 mL) and acetone (5 mL) to remove any remaining impurities and stored for reuse. The reusability of the catalyst was tested across four successive cycles. The catalyst maintains high activity for three consecutive reuses; a more noticeable decrease is observed from the fourth reuse, likely due to partial surface fouling with organic material under solvent-free, high-temperature conditions (Fig. 4).

**Scheme 4:**
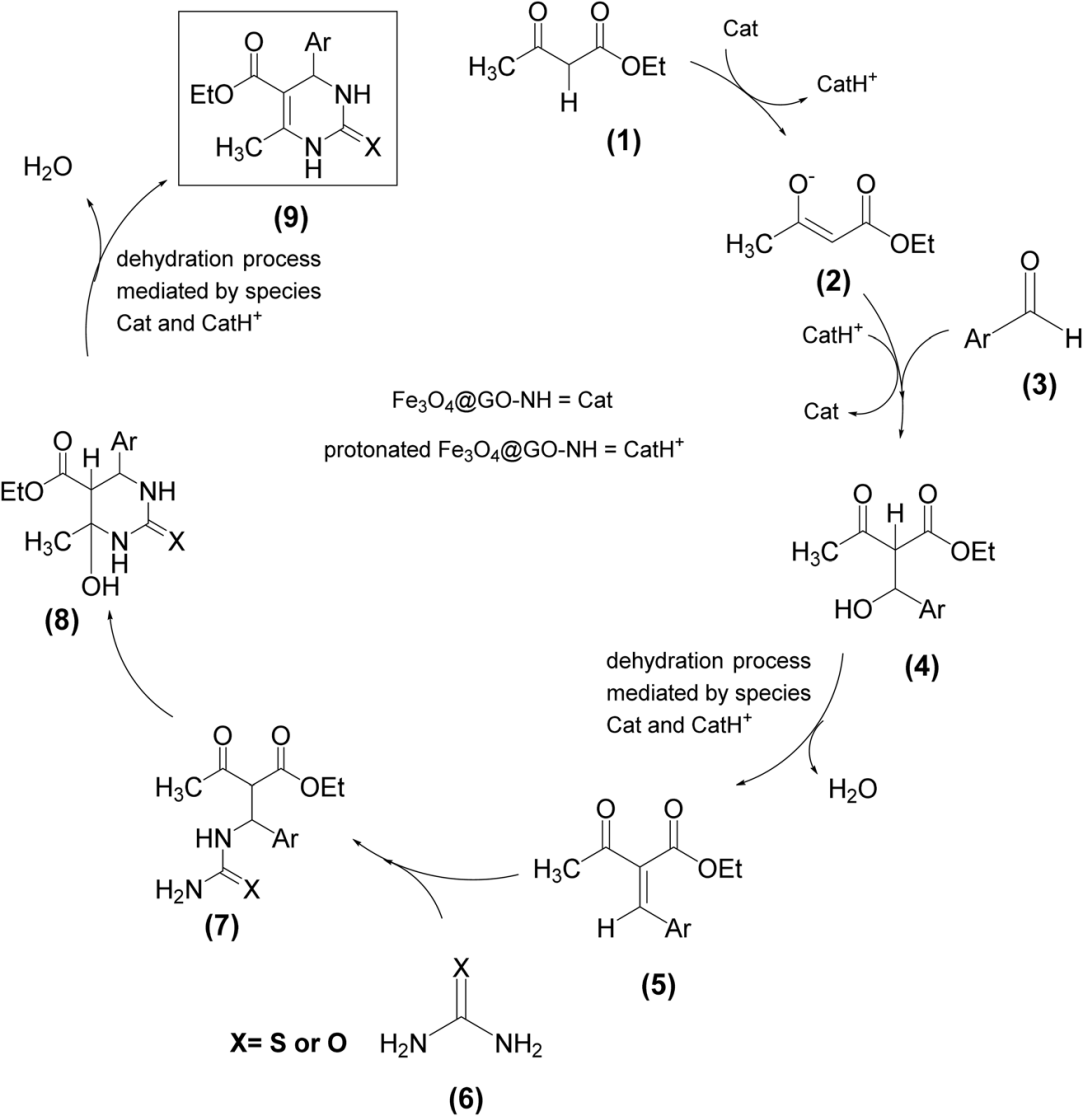
Proposed mechanism of the Biginelli reaction catalyzed by Fe_3_O_4_@GO-NH.

Considering the structural features of Fe_3_O_4_@GO-NH, its catalytic efficiency in the Biginelli reaction can be attributed to its ability to act as a Lewis (due to Fe^3+^ or Fe^2+^ ions present in Fe_3_O_4_) or Brønsted acid (due to the protonated piperazine moiety) and Brønsted base (due to the piperazine moiety) in multiple protonation and deprotonation reaction steps. Scheme 4 illustrates our proposed mechanism for the Biginelli reaction catalyzed by Fe_3_O_4_@GO-NH, depicting its dual role as both an acid and a base. Initially, it acts as a Brønsted base by deprotonating and activating β-ketoester 1, making it more nucleophilic for attack on aldehyde 3 activated by Fe^3+^ ion, Fe^2+^ ion or the protonated Fe_3_O_4_@GO-NH (CatH^+^). Moreover, the Lewis/Brønsted acidic and basic sites of the catalyst can promote the protonation and deprotonation steps in the dehydration processes, forming intermediate 5 and product 9.

#### 3.3.3. Comparison of efficiency with that of catalysts reported in the literature

A wide range of homogeneous and heterogeneous catalysts has been explored for the Biginelli reaction, yet many of these methods still encounter drawbacks such as elevated temperatures, long reaction times, or moderate yields. For instance, MCM-41-HClO_4_ in methanol at 80 °C required 4 h to afford a 76% yield,^45^ while HTMA in acetic acid at 100 °C gave a similar yield of 75% after 5 h.^46^ H-MOR use in toluene at 110 °C required 10 h, resulting in only 60% yield.^47^ Some solvent-free protocols have also been attempted: nano-γ-Fe_2_O_3_@SiO_2_ and bulk-Fe_2_O_3_–SO_3_H achieved 20% and 65% yields, respectively, within just 3 min at 60 °C,^48^ although their efficiency remained limited (Table 3). By contrast, our Fe_3_O_4_@GO-NH catalyst under solvent-free conditions at 130 °C afforded an excellent 97% yield in only 75 min. In addition, the catalyst can be magnetically separated and reused over multiple cycles with negligible activity loss, offering a greener, more efficient, and more sustainable alternative than many previously reported catalytic systems.

**Table 3:** Representative literature examples of catalysts and conditions reported for the Biginelli product 2a under solvent-free conditions

Entry	Temp. (°C)	Time (min)	Catalyst	Yield (%)	Ref.
1	120	50	Nano-ZrO_2_ sulfuric acid	89	45
2	120	90	H_4_[PVW_11_O_40_]/activated natural clay	91	46
3	100	60	Fe_3_O_4_@SiO_2_@Trz-Cu (copper 1,2,3-triazole complex)	82	47
4	130	30	Bentonite/PS-SO_3_H (polymer-supported sulphonic acid)	90	48
5	130	75	Fe_3_O_4_@GO-NH	97	This work

## 4. Conclusion

In conclusion, the development of Fe_3_O_4_@GO-NH as a heterogeneous catalyst for the Biginelli reaction represents a significant advancement in sustainable catalysis. Fe_3_O_4_@GO-NH catalyst was successfully synthesized and analyzed by FT-IR, TGA, SEM/EDX. The catalyst exhibited remarkable catalytic activity under solvent-free conditions with easy isolation and excellent reusability. By optimizing reaction parameters, 32 Biginelli products were efficiently synthesized. The optimal conditions involved a solvent-free environment at 130 °C for 75 min, utilizing 0.015 g of catalyst Fe_3_O_4_@GO-NH. Furthermore, the catalyst showed superior catalytic performance compared to other catalysts, providing high yields and ease of recyclability. The approach demonstrated here offers a promising, eco-friendly alternative for the Biginelli reaction, with significant potential for broader applications in organic synthesis.

## Author contributions

Esmail Rezaei-Seresht: writing – review & editing, supervision, conceptualization. Samira Cheshak: writing – original draft, visualization, validation, investigation. Faezeh Jalambadani: writing – original draft, investigation, visualization, data curation. Fatemeh Tafazzoli Gazkoh: writing – original draft. Behnam Mahdavi: supervision.

## Conflicts of interest

There are no conflicts to declare.

## Supplementary Material

RA-016-D5RA05063D-s001

## Data Availability

Data sharing is not applicable to this article as no new data were created or analyzed in this study. Supplementary information (SI) is available. See DOI: https://doi.org/10.1039/d5ra05063d.
