# Supplementary material for: Piperazine-grafted magnetic graphene oxide as a sustainable heterogeneous catalyst for solvent-free Biginelli reaction
Source: RSC Adv. 2026 Feb 24;16(12):10735–49. doi: 10.1039/d5ra05063d (PMC12930250; doi:10.1039/d5ra05063d)
Supplement: RA-016-D5RA05063D-s001 [file RA-016-D5RA05063D-s001.pdf]

Supplementary Information (SI) for RSC Advances.
This journal is © The Royal Society of Chemistry 2026

Supplementary Information

Piperazine-Grafted Magnetic Graphene Oxide as a Sustainable Heterogeneous Catalyst for Solvent-Free Biginelli Reaction

Esmail Rezaei-Seresht *, Faezeh Jalambadani, Samira Cheshak, Behnam Mahdavi, Fatemeh Tafazzoli Gazkoh

Department of Chemistry, School of Sciences, Hakim Sabzevari University, Sabzevar, Iran

*Corresponding author. Tel./fax: +98 44013516.
E-mail address: e.rezaei@hsu.ac.ir

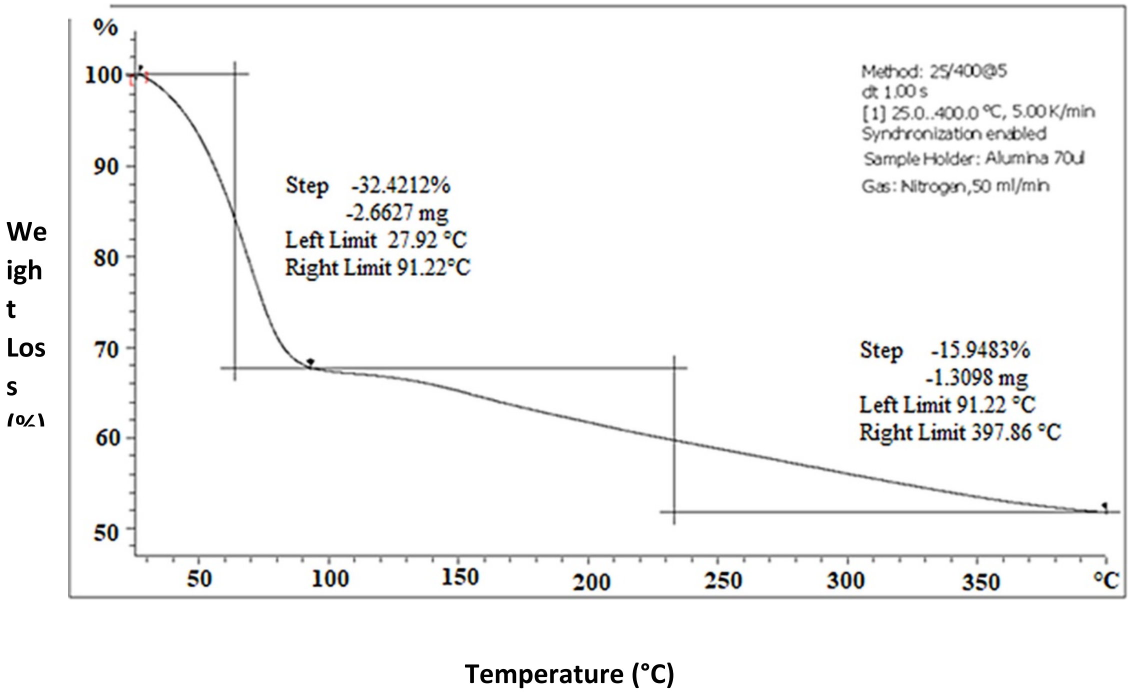

Figure 1. TGA curve of catalyst Fe_3O_4@GO-NH.

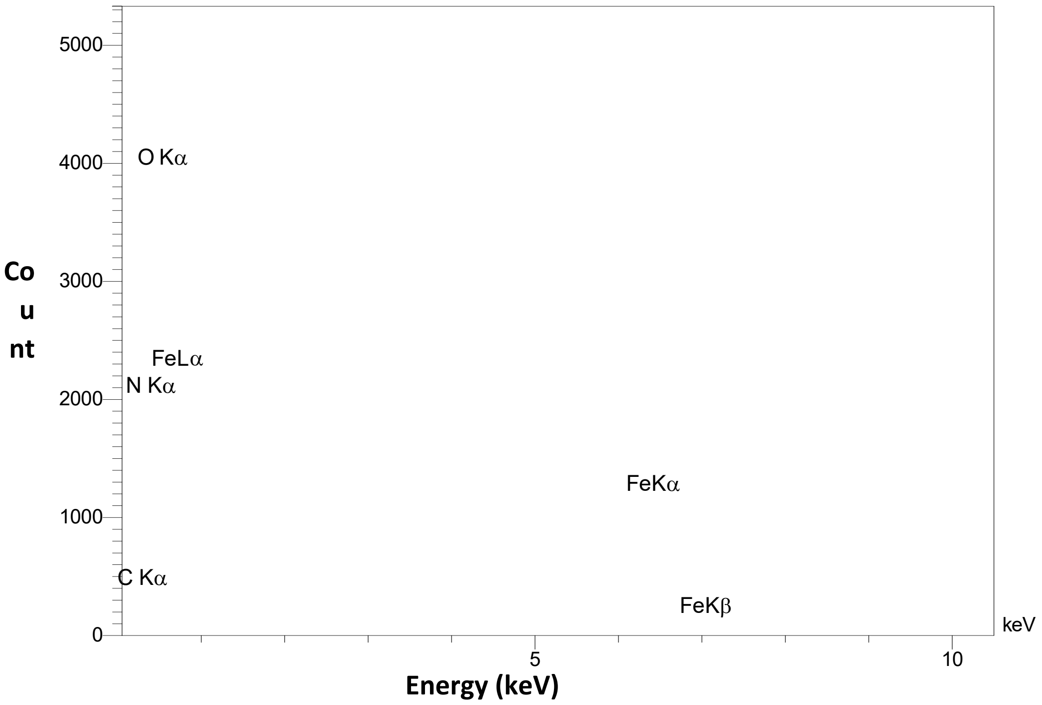

Figure 2. EDX spectrum of catalyst Fe_3O_4@GO-NH.

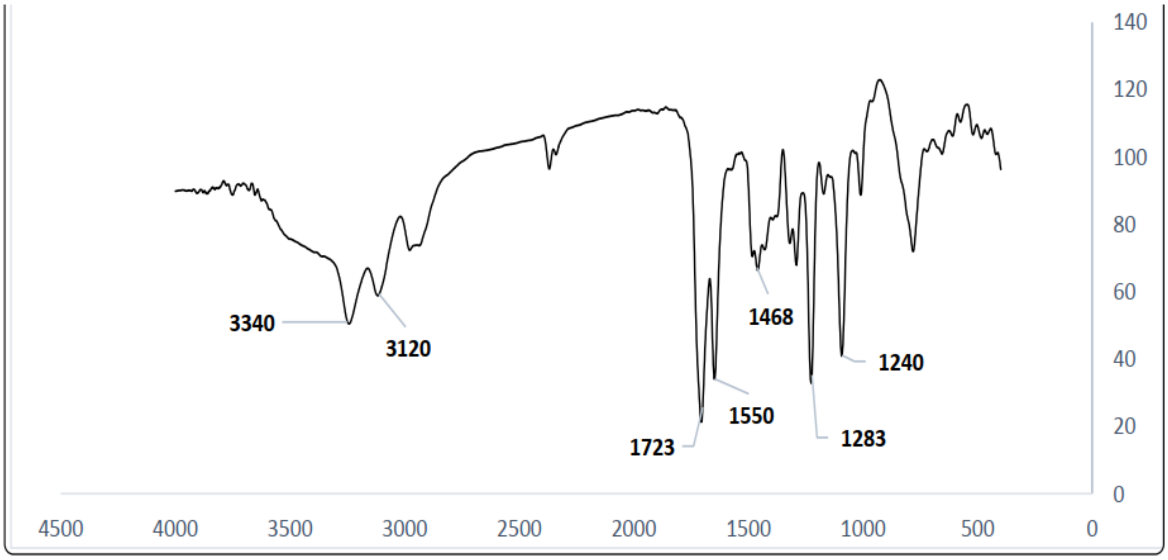

Figure 3. FTIR spectrum of Ethyl 4-(4-bromophenyl)-6-methyl-2-oxo-1, 2, 3, 4-tetrahydropyrimidine-5-carboxylate (**2b**).

Wavenumber (cm^{-1})

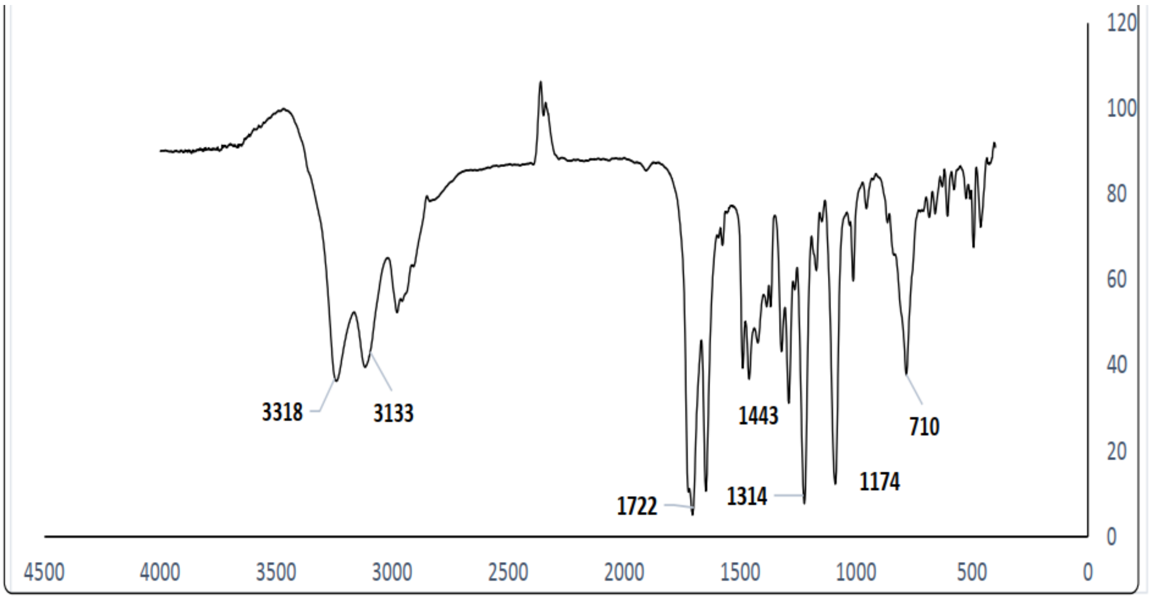

Figure 4. FTIR spectrum of Ethyl 4-(4-chlorophenyl)-6-methyl-2-oxo-1, 2, 3, 4-tetrahydropyrimidine-5-carboxylate (**2c**).

Wavenumber (cm^{-1})

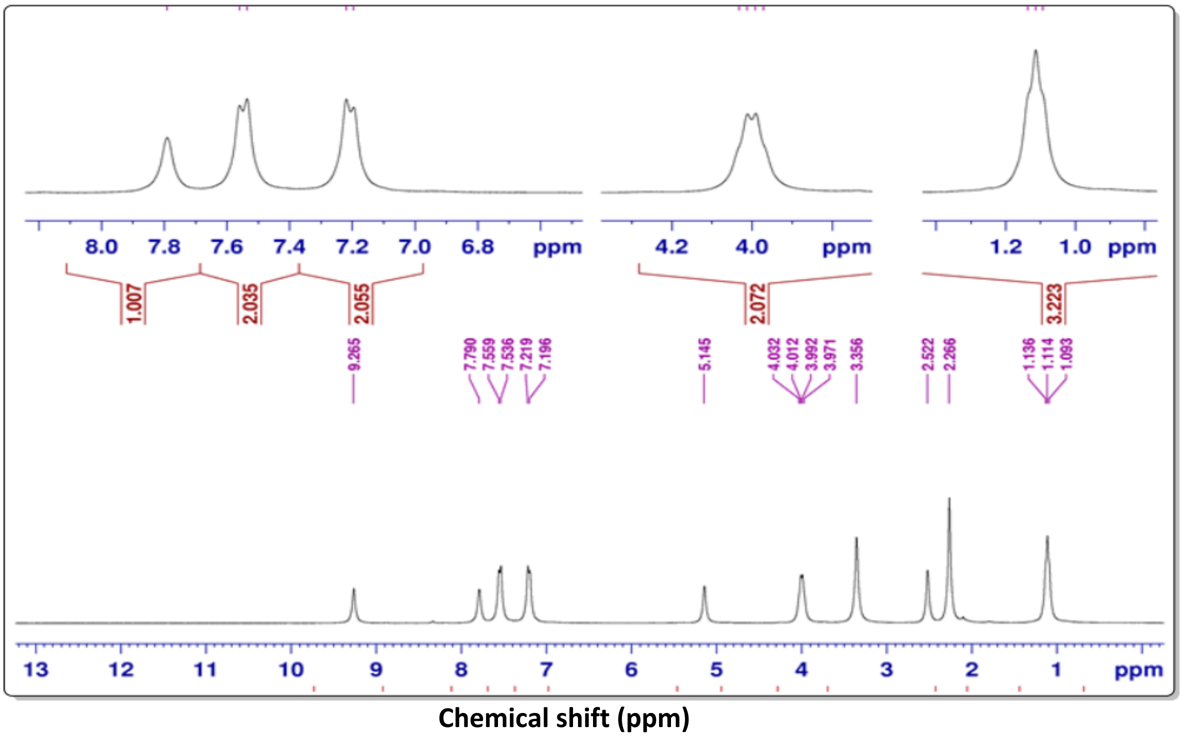

Figure 5. ^{1}H NMR spectrum of Ethyl 4-(4-bromophenyl)-6-methyl-2-oxo-1, 2, 3, 4-tetrahydropyrimidine-5-carboxylate (**2b**).

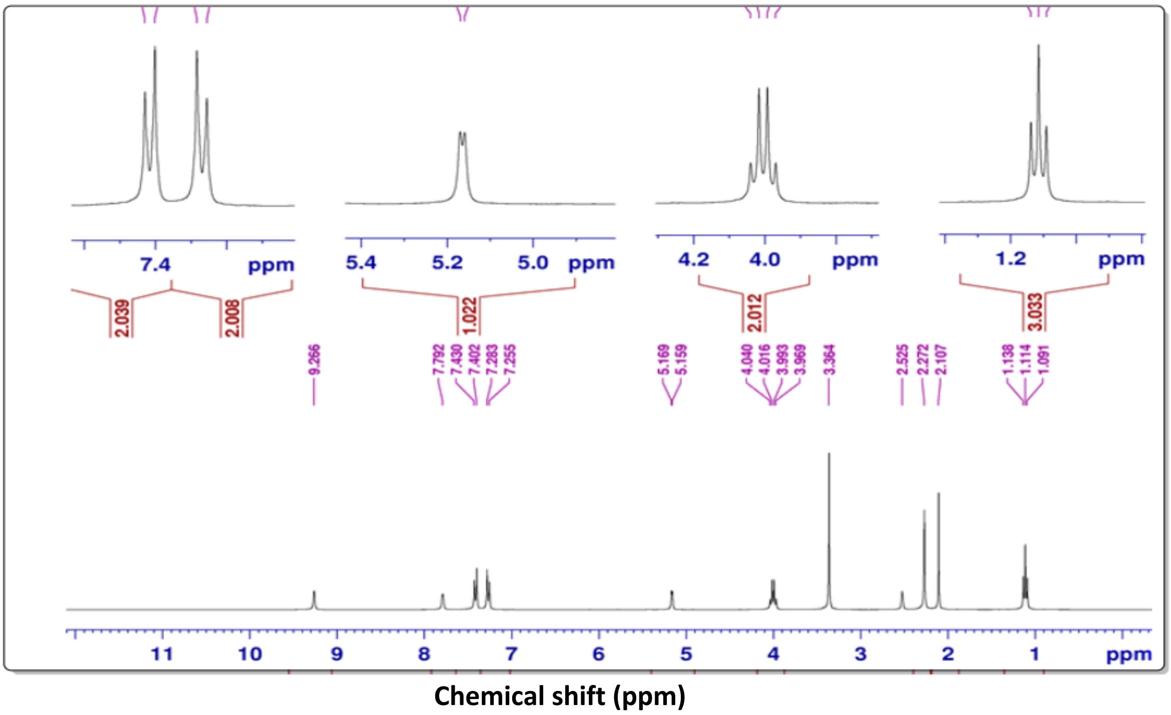

Figure 6. ^{1}H NMR spectrum of Ethyl 4-(4-chlorophenyl)-6-methyl-2-oxo-1, 2, 3, 4-tetrahydropyrimidine-5-carboxylate (**2c**).

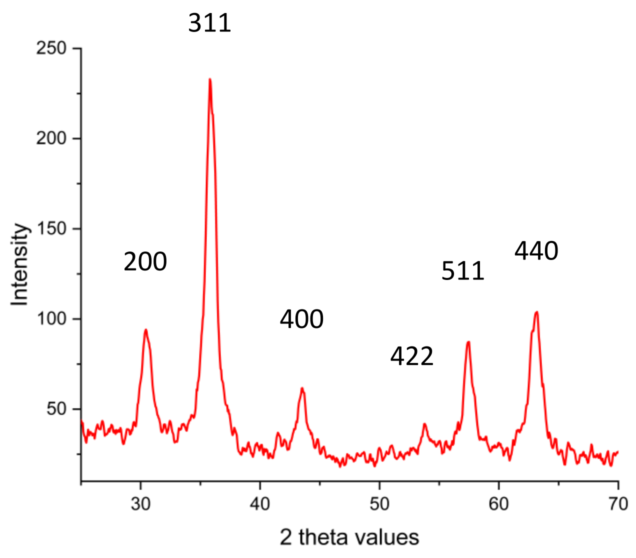

Figure 7. XRD pattern of Fe_3O_4. The diffraction peak positions match well with JCPDS 00-003-0863 for magnetite.

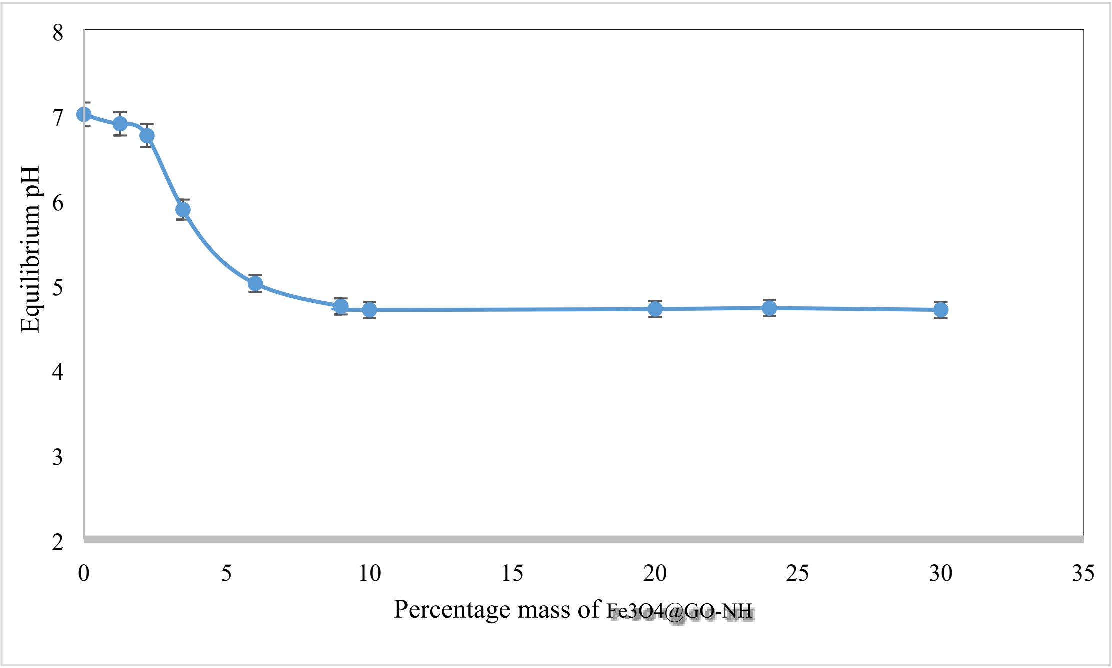

Figure 8. Influence of Fe_3O_4@GO-NH mass fraction on the equilibrium pH of the suspension in 0.01 M KCl at 25 ± 2 °C. The plateau at pH 4.70 represents the PZC value. Error bars indicate standard deviation of the mean (±SD).

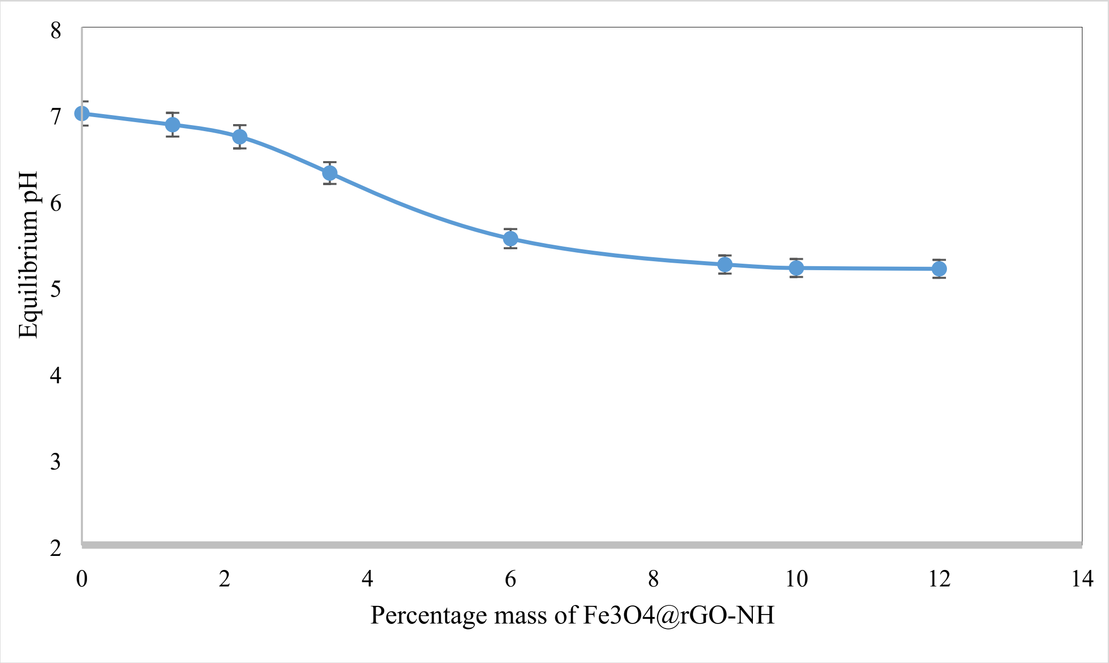

Figure 9. Influence of Fe_3O_4@rGO-NH mass fraction on the equilibrium pH of the suspension in 0.01 M KCl at 25 ± 2 °C. The plateau at pH 5.21 represents the PZC value. Error bars indicate standard deviation of the mean (±SD).
